# Silicon-based MEMS/NEMS empowered by graphene: a scheme for large tunability and functionality

**DOI:** 10.1038/s41378-025-00960-0

**Published:** 2025-06-09

**Authors:** Mengqi Fu, Zhan Shi, Bojan Bošnjak, Robert H. Blick, Elke Scheer, Fan Yang

**Affiliations:** 1 Fachbereich Physik, Universität Konstanz, 78457 Konstanz, Germany; 2 Department of Mechanics, Key Laboratory of Soft Machines and Smart devices of Zhejiang Province, Zhejiang University, 310058 Hangzhou, China; 3 Center for Hybrid Nanostructures, Universität Hamburg, 22761 Hamburg, Germany; 4 Dynamic Precision Micro&Nano Sensing Technology Research Institute, Chongqing, 400030 Chongqing, China

**Keywords:** NEMS, Sensors

## Abstract

Integration of graphene in silicon-based micro-/nanoelectromechanical systems (MEMS/NEMS) marries the robustness of silicon-based materials with the exceptional physical properties of graphene, drastically enhancing the system’s regulation performance which now is key for many advanced applications in nanotechnology. Here, we experimentally demonstrate and theoretically analyze a powerful on-chip integration principle consisting of a hybrid graphene/silicon nitride membrane with metallic leads on top that enables an extremely large static and dynamic parameter regulation. When a static voltage is applied to the leads of the integrated structure, a spatially confined localized electrothermomechanical (ETM) effect results in ultra-wide frequency tuning, deformation (buckling transition) and regulation of the mechanical properties. Moreover, by injecting an alternating voltage to the leads, we can excite the resonator vibrating even far beyond its linear regime without a complex and space consuming actuation system. Our results prove that the scheme provides a compact integrated system possessing mechanical robustness, high controllability, and fast response. It not only expands the limit of the application range of MEMS/NEMS devices, but also enables the further miniaturization of the device.

**Figure:**
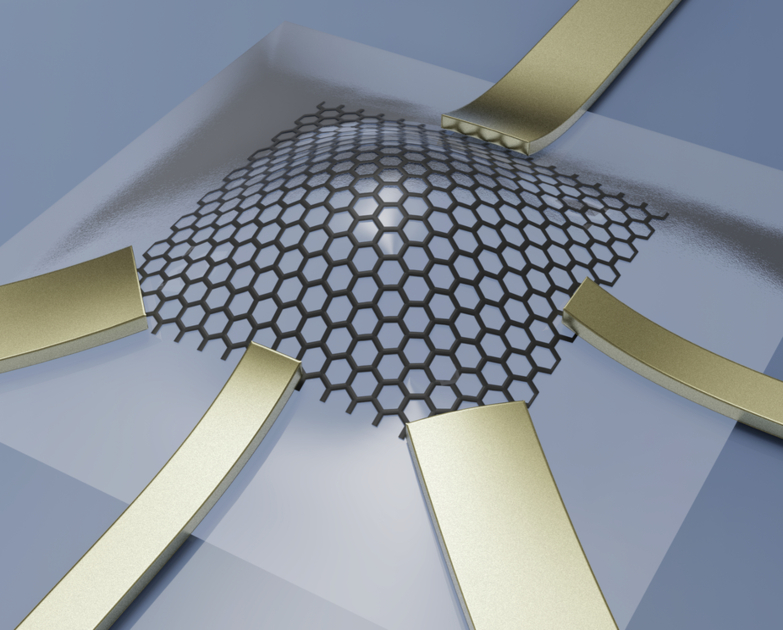
The graphene integrated MEMS/NEMS empowered by graphene: a scheme for strong enhancements of tunability and functionality of silicon based device device consists of a hybrid graphene/silicon-nitride membrane with metallic leads that enables ultra-wide frequency tuning, spatial deflection, mechanical properties tuning and on-surface actuation.

The graphene integrated MEMS/NEMS empowered by graphene: a scheme for strong enhancements of tunability and functionality of silicon based device device consists of a hybrid graphene/silicon-nitride membrane with metallic leads that enables ultra-wide frequency tuning, spatial deflection, mechanical properties tuning and on-surface actuation.

## Introduction

Silicon-based MEMS/NEMS play an essential role in sensing, for switching, actuation, and filtering applications^1–5^, also in fundamental research such as optomechanics^6–8^, nonlinear dynamics^9–11^, and other fields of physics^12,13^. Regulating the mechanical properties of MEMS/NEMS over a large range is crucial for a variety of applications, e.g., for broad-band eigenfrequency tuning, multimode sensing, frequency stabilization and parametric driving^3,14,15^. Moreover, the programmable regulation of spatial symmetry enables application as nano-actuators^4,16^ for morphology changes applied in flexible devices^17^ like mechanical switches^3^, and nanorobotics^18^. However, due to the high rigidity of silicon-based materials, enhancing the tuning ability of established regulation methods is a challenge in MEMS/NEMS devices, particularly in on-chip tuning structures that favor device miniaturization.

Among the common regulation methods, the functional structures employing ETM effects have been shown to be an effective tool for tuning both the dynamic properties and morphology^19–22^. The archetypical example for an ETM structure is the multilayered Timoshenko beam, i.e., a bilayer structure composed of different materials and involving both bending and shear deformations^23^ that is the building block of standard thermally activated switches. Integrating on-chip thermal regulation structures into nanomechanical devices usually requires complex design and fabrication techniques. Taking advantage of their favorable mechanical and electronic properties, often graphene or other two-dimensional (2D) materials are utilized as functional components in NEMS devices^24,25^. Here we show that the ultra-low mass and the contact resistance of graphene (G) with metal (M) leads are beneficial to use them as on-chip thermal regulation structures in silicon-based MEMS/NEMS devices.

In this work, we present a scheme for extreme enhancements of tunability and functionality of silicon based MEMS/NEMS device by taking an example of a metal-graphene-silicon nitride (MGS) hybrid structure combined with an ETM tuning mechanism to mechanically deform a freestanding silicon nitride (SiN) membrane resonator. It achieves the controlling of the eigenfrequency and the nonlinearity of the mechanical response over an extremely wide range with high efficiency. The monolayer G sandwiched between SiN and M electrodes acts as an electric conducting channel as well as an interface for inducing ETM effects into the SiN–M system, which simplifies the design of the regulation structure. By leveraging the ultralow mass and high transparency of G^15,26^, some excellent mechanical (e.g., low damping rate) and optical properties of the SiN layer are largely preserved. Additionally, the high electron mobility of G enables operation at high frequencies. Furthermore, we demonstrate that the MGS device features on-surface vibration excitation with the capability of controlled symmetry breaking. We discuss the ETM actuation mechanism and quantitatively demonstrate the excellent performance of the symmetry breaking control and on-surface actuation of the MGS resonator.

## Results and discussion

### Design concept of the MGS device

**Fig. 1:**
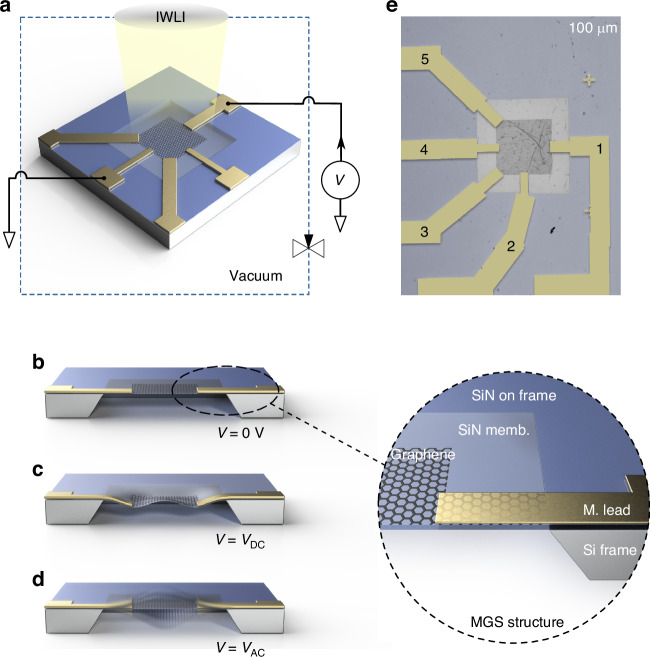
Experimental scheme and demonstration of the concept. **a** A 3D sketch of the MGS device, the electric circuit, and the IWLI. The graphene area is fixed by the leads, the SiN membrane holds the G and M structures and is clamped by a Si frame. **b**–**d** Simplified cross-section sketches of the MGS device (only one pair of leads: 1 and 4, see **e**) for different conditions: **b** plain surface for *V* = 0, with zoom into one of the edges, where each element is labeled. The metal lead on top of the SiN membrane is illustrated semitransparently to show the G underneath. **c** Static deformation with *V* = *V*_DC_, and **d** periodic vibration with *V* = *V*_AC_. **e** Optical micrograph of the fabricated MGS device. The metal leads are labeled from 1 to 5

The designed MGS resonator, as depicted in Fig. 1a, is based on a silicon chip that carries a fully-clamped freestanding SiN membrane. To induce ETM effects locally on the membrane, a graphene-metal (G-M) structure is placed on the SiN membrane. The patterned monolayer G is located centrally on the SiN membrane, serving as the conductive channel. The M (Ti/Au, 25/25-nm thick) leads are evaporated on top for providing electrical contacts and for ETM actuation. The cross section of the MGS resonator is presented in Fig. 1b.

The designed ETM actuation scheme is based on the force between the M leads and the SiN membrane induced by their different thermal expansion coefficients. Hence we choose a material combination that features a large gradient of thermal expansion coefficients from the top Au to the bottom SiN (*α*_Au_ = 14.2 ⋅ 10^−6^/K, *α*_Ti_ = 7.6 ⋅ 10^−6^/K, and ${\alpha }_{{\rm{SiN}}}=3.2\cdot 1{0}^{-6}$/K). Since the thermal expansion itself will be caused by Joule heating, we take advantage of the high electrical conductance of the G and the relatively high contact resistance of the M-G interface to localize the Joule heating to the small overlap area between G and the M leads. The local voltage drop and related dissipation at the contacts causes the ETM effect that tunes the mechanical properties of the device, as shown in Fig. 1b. It can both statically deflect the SiN membrane by applying a direct voltage *V*_DC_, as shown in Fig. 1c, and dynamically excite the vibration of the membrane by applying an alternating voltage *V*_AC_, as shown in Fig. 1d.

The device under study here as proof-of-principle is shown in Fig. 1e. A 0.5-mm thick (100) silicon wafer with 110 nm LPCVD SiN dual-side grown, undergoes wet-etching in KOH for SiN membrane fabrication, measuring as 495 × 512 μm^2^ in lateral dimension and 110 nm in thickness. The G is LPCVD-grown and wet-transferred onto the SiN, extending to the Si frame. A negative resist is spin-coated and patterned via electron beam lithography on G, followed by oxygen plasma etching to define a 300 × 300 μm^2^ G channel (the mass of the G is approximately 6.7 × 10^−14^ kg) and the extended contact area (same size as the M electrode on top to ensure an even heating process and a well-defined contact resistance) of the G. The resist is removed in hot acetone, and M leads are patterned with positive resist and Ti/Au electron beam evaporation process. After the lift-off, the device fabrication is finalized. The sample under study here features five leads, labeled 1–5. The size of lead 1 and 4 are 60 × 100 μm^2^ and 50 × 100 μm^2^, respectively. The resistance of the G is about 1.65 k*Ω* and the contact resistance to the M electrode *R* is about 5–10 times larger, varying from lead to lead and the applied voltage. For more details, see the Supporting Information (SI)^27^.

The advantages of low mass, high optical transparency, relatively high conductance and high resistance to the M are all optimally included in monolayer G. First, the high flexibility, small bending stiffness, and ultra-low mass of the G sheet are beneficial for the mechanics. Since the bending stiffness and mass of the G are negligible compared to the ones of the SiN membrane^28,29^, it provides a quasi-free end for the M leads on the surface. The mechanical properties of this MEMS/NEMS device are dominated by the SiN membrane and the ETM effects are regulated by the properties of the MGS structure. Second, thanks to its high conductance and the contact resistance to M, G can serve as conducting path between the electrodes, and the spatially confined contact resistance to the M serves to locally generate heat at a predefined position. Last but not least, the high optical transparency (97.7%^30^) of G ensures that the optical properties of the SiN are minimally affected, which is beneficial for the application in fields such as optomechanics. We note that wrinkles of the G sheet should only play a quantitative, but not a qualitative role for the functionality of the device, e.g., they slightly increase the contact resistance in the area of the MGS structure. In the SI we present control experiments with M electrodes alone without G in the SI^27^.

### Static spatial symmetry control

**Fig. 2:**
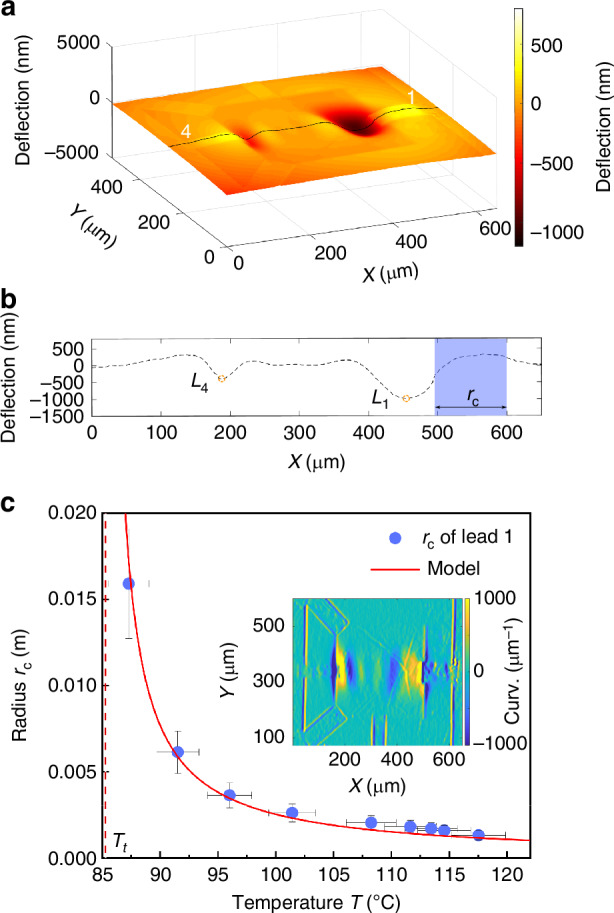
Experimental measurement of controlled static deformation and extracted curvature radius *r*_*c*_ of the deflection. **a** IWLI-captured image of the spatial deflection of the MGS resonator with *V*_DC_ = 2.9 V. **b** Deflection profile of the resonator at the position on the membrane surface indicated by the black line in (**a**). The two maxima of the deflected SiN membrane marked as *L*_1_ and *L*_4_ correspond to the positions of the open ends of leads 1 and 4, respectively. The spatial range used for calculating *r*_*c*_ of the MGS structure is indicated by the blue-shaded area. **c** Extracted *r*_*c*_ plotted as function of the corresponding temperature (heated up by *V*_DC_ from 2.65 V up to 2.9 V, blue dots), the calculation of the bilayer model is plotted as red solid line. The red-dashed line showing the buckling transition temperature *T*_*t*_. The inset shows the curvature distribution extracted from the IWLI image obtained for *V*_DC_ = 2.9 V

We use leads 1 and 4 for applying the *V*_DC_. Up to approximately 2.5 V, the MGS structure remains flat. An out-of-plane static deflection of the MGS device occurs when *V*_DC_ exceeds approximately 2.5 V, corresponding to the scheme in Fig. 1e. The amplitude and the curvature of the static deflection increase proportionally to *V*_DC_, as shown in the SI^27^. A typical 3D image of the static spatial deflection captured by imaging white light interferometry (IWLI) at *V*_DC_ = 2.9 V with a line cut parallel to the *x*-axis is shown in Fig. 2a, b. The strongest downward deflections of the device are located around the free ends of leads 1 and 4. The MGS structures are convexly bent upwards, indicated by the bright yellow structures in Fig. 2a. The maximum deflection, beyond 1300 nm, occurs around the edge of lead 1 (point *L*_1_ ≈ 450 μm in the *x*-axis in Fig. 2b). The images under different *V*_DC_ show similar bending patterns but different amplitudes of deflection, as shown in the SI^27^. When the *V*_DC_ is switched off, the system recovers its original flat state, proving that the deflection is reversible over this range of *V*_DC_.

The very large spatial deflection of the MGS resonator under *V*_DC_ can be fully described by the buckling transition induced by unequal thermal expansion of the M leads and the SiN membrane, as explained below^31,32^. When the MGS structure senses heat, the M layer elongates and generates a force component perpendicular to the SiN plane, henceforth referred to as buckling force, arising from the directly exerted linear force component(sliding is prevented). After a certain threshold, the MGS structure spontaneously bends, acting as a typical Timoshenko bilayer structure^23,27^. The M leads bend downwards at the free end, resulting in a detectable curvature and deformation of the SiN layer.

We calculated the relationship between Joule heating and the curvature radius *r*_*c*_ for a simplified model representing one side of the MGS resonator (e.g., lead 1) as a uniformly heated bilayer cantilever with a stress constantly applied on the free end. Since the Ti and Au layers have similar thickness, we use a simplified metal film model with averaged material parameters (thermal properties). In addition, we assume that the majority of the heat is generated at the interface between the M lead and the G. In the thermal steady state of the structure, the temperature profile is determined by the Joule heat generation and the heat dissipation to the environment. From the geometry and the thermal conductivities of the components, we estimate that the dominant dissipation component is the heat flow from the heated M electrode through the membrane to the frame due to its high thermal conductivity and large cross section, while the heat flow in the lateral direction of the SiN layer as well as radiation losses are several orders of magnitude smaller. Neglecting these other contributions, we estimate the temperature at different *V*_DC_ with a simple one dimensional Fourier law:

1 $$T={T}_{0}+\frac{{L}_{{\rm{M}}}{P}_{{\rm{M}}}}{\kappa {W}_{{\rm{M}}}{h}_{{\rm{M}}}},$$

where *T*_0_ is the starting temperature (here: room temperature), *L*_M_, *W*_M_, and *h*_M_ denote the length, width and thickness of the M lead acting as heat source. *κ* is the thermal conductivity of the M lead, and *P*_M_ is the power load to the structure with the M-G contact resistance *R*. Here, considering the resistance contribution of the G and the contact resistance, *P*_M_ is calculated as 42.5% of the total applied power *P* = *IV*. This value takes into account that in total about 85% of the electrically dissipated power contributes to the heating of the membrane and that both leads contribute equally to the heating. This estimation yields a temperature difference between the heated part of the M electrode and the heat bath on the order of 100 K for the voltages used here. We verified the temperature gradient along the M electrode, the order of magnitude of the temperature difference and the effective length *L*_*M*_ by measuring the Raman shift of hexaboronnitride cover layers on a test sample, as described in the SI^27^. Furthermore, we performed finite-element simulations taking into account a more realistic two-dimensional geometry to confirm the size of the temperature gradient and the linearity between the local temperature at the heating point and the heating power, see the SI^27^ for more details.

The curvature of the MGS at different temperature *T* follows the general equation:

2 $${r}_{c}=\frac{({h}_{{\rm{M}}}+{h}_{{\rm{SiN}}})\left[3{(1+{h}^{{\prime} })}^{2}+(1+{h}^{{\prime} }{E}^{{\prime} })\left({h}^{{\prime} 2}+\frac{1}{{h}^{{\prime} }{E}^{{\prime} }}\right)\right]}{(6\varepsilon \cdot (T-{T}_{0})-{\sigma }_{{\rm{SiN}}}/{E}_{{\rm{SiN}}}){(1+{h}^{{\prime} })}^{2}},$$

where ${h}^{{\prime} }={h}_{{\rm{M}}}/{h}_{{\rm{SiN}}}$, ${E}^{{\prime} }={E}_{{\rm{M}}}/{E}_{{\rm{SiN}}}$, and *ε* = *α*_M_ − *α*_SiN_. Here, ${h}_{{\rm{SiN}}}$ and *h*_M_ represent the thicknesses of SiN and Au/Ti, respectively. ${E}_{{\rm{SiN}}}$, *E*_M_, ${\alpha }_{{\rm{SiN}}}$, and *α*_M_ represent the Young’s moduli and thermal expansion coefficients of the two layers, respectively. ${\sigma }_{{\rm{SiN}}}$ represents the residual stress of SiN. For more detailed information, we refer to the SI^27^.

The *r*_*c*_ values extracted from the experiment as a function of temperature *V*_DC_ from 2.5 V up to 2.9 V, applied between leads 1 and 4 are shown as blue dots in Fig. 2c, while the calculated *r*_*c*_ using Eq. (2) is shown as a red solid line. The curvature distribution of the membrane at *V*_DC_ = 2.9 V is shown in the inset of Fig. 2c for a full visualization of the spatial deflection. The results demonstrate that the calculated *r*_*c*_ matches the experimental results well without any free fitting parameter, supporting the notion that the strong mismatch of the thermal expansion coefficients of the different materials in the MGS structure is the origin of the static deformation, which can be engineered and predicted accurately. A finite element analysis also proves the consistency, for details see the SI^27^.

The model also provides access to the buckling transition temperature, required to initiate the spatial deflection, ${T}_{t}=({\sigma }_{{\rm{SiN}}}/6\varepsilon {E}_{{\rm{SiN}}})+{T}_{0}\simeq$ 85 °C (*V*_DC_ ≳ 2.5 V). *T*_*t*_ is obtained from Eq. (2) as the temperature at which the first factor in the denominator vanishes and *r*_*c*_ hence diverges. At *T*_*t*_, marked in Fig. 2c, the ETM induced force is exactly compensated by the local residual stress. Below *T*_*t*_, no spatial deflection is detectable. More importantly, the model reveals how *T*_*t*_ can be engineered by designing the material properties and geometry, including residual stress, Young’s modulus, and the thermal expansion coefficient of each component of the hybrid structure.

In general, creating such strong and controlled static deformation in SiN membranes is difficult to achieve due to their high prestress and stiffness, especially when using on-chip structures such as dielectric gates. Most impressively, the ETM-induced strain of the MGS device can be extremely large and even break the SiN membrane, as demonstrated in the SI^27^, in the section “Destructive test”. As the example shows, above a certain power load, the device breaks. However, they do not break electrically, but mechanically, above a certain deformation amplitude.

### Regulation of dynamic properties

**Fig. 3:**
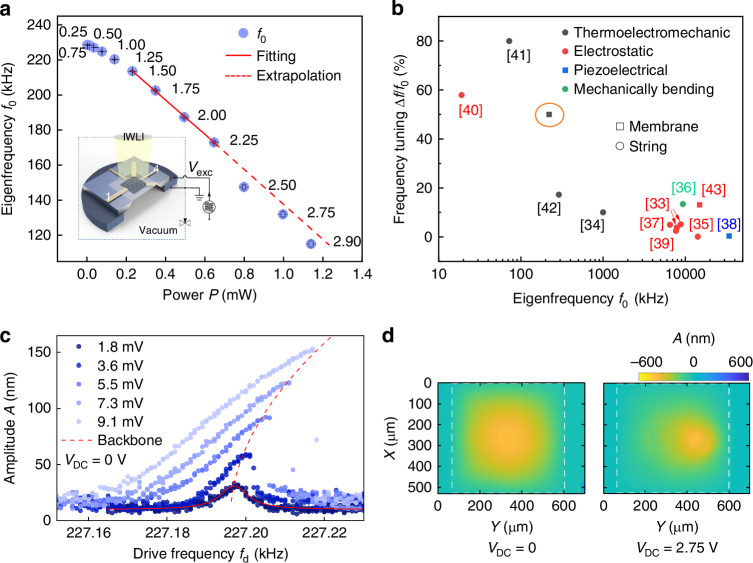
Experimental demonstration of dynamic properties tuning. **a** Tuning of the linear eigenfrequency *f*_0_ of the (1,1) mode with increasing DC power *P* loaded between leads 1 and 4. A linear fit is applied to the data with 0.2 ≤ *P* ≤ 0.64 mW and plotted as a red-dashed line extended to the full data range. The inset illustrates the measurement scheme for obtaining the linear mechanical response of the MGS resonator. An AC sinusoidal drive signal *V*_exc_ is sent into the piezo attached to the bottom of the chip to drive the resonator into vibration. Part of the structure is cut out to show the 3D spatial arrangement. The numbers next to the data points indicate the corresponding *V*_DC_ in *V*. **b** Reported tuning capabilities comparing different approaches of silicon-based MEMS/NEMS systems. Round (square) symbols represent data from string (membrane or plate) resonators^33,48–57^. The value of our present research is marked by an orange circle. **c** Nonlinear mechanical vibrational response curves of the MGS resonator with *V*_exc_ applied to the piezo from 1.8 mV up to 9.1 mV with *V*_DC_ = 0 V. **d** Shows the IWLI-captured vibrational pattern of the (1,1) mode at phase *ϕ* = 90° under *V*_DC_ = 0 V (left) and 2.75 V (right), respectively. The *V*_DC_ is applied to leads 1 and 4. The MGS device is driven by the piezo with *V*_exc_ = 0.5 V and the *f*_*d*_ is slightly above the respective *f*_0_

We now discuss the dynamic property tuning utilizing the ETM effect. The dynamic properties of the MGS device, such as the eigenfrequency *f*_0_, the damping rate, the nonlinearity and the vibrational pattern, can be characterized from their vibration response. In our setup, we solidly attach the MGS resonator onto a piezo ring (serving as drive system), as shown in the inset of Fig. 3a. The mechanical response of the driven motion is measured optically by IWLI. The drive frequency *f*_*d*_ at which the maximal vibration amplitude in the linear response is observed is identified as *f*_0_. By fitting the linear response with a Lorentzian function and the nonlinear response with Duffing model, respectively, the damping rate (and thus the quality factor *Q*) as well as the cubic nonlinearity can be quantitatively extracted. For instance, Fig. 3c shows a series of mechanical response curves (*V*_DC_ = 0 V) for the (1,1) mode of the MGS resonator measured by IWLI. With *V*_exc_ ranging from 1.8 mV to 9.1 mV, the response curves vary from a Lorentzian shape to a Duffing-type shape and the backbone trace is indicated by a red-dashed line. The *f*_0_ and *Q* are determined as 227.196 kHz and 2.8 × 10^4^ from the response curve with *V*_exc_ = 1.8 mV in Fig. 3c). The Duffing nonlinearity is extracted as 3.5 × 10^22^ m^−2^s^−2^ (see SI^27^).

We first monitor the frequency tuning effect by characterizing the *f*_0_ shift of the driven mode. Figure 3a shows the dependence of the *f*_0_ of the ground mode ((1,1) mode) on the power load *P* with *V*_DC_ applied between leads 1 and 4. *f*_0_ decreases roughly linearly for increasing *P* up to 0.64 mW (*V*_DC_ = 2.25 V), as shown by the red dashed line. The damping rate remains almost unchanged, see the SI^27^. At *P* ≃ 0.79 mW (*V*_DC_ ≃ 2.50 V), *f*_0_ shows a jump-like decrease and then follows the linear trend again with very similar slope as before the jump. This jump occurs at the power load of the onset of spatial deflection at *T* = *T*_*t*_ ≃ 85 °C. Upon increasing *P* to 1.16 mW (corresponding to *V*_DC_ = 2.9 V), *f*_0_ decreases to 110 kHz, indicating a tuning of more than 50%. The offset in the linear tendency reveals the additional effect of the buckling on the geometric properties and intensifies the tuning of *f*_0_ by *P*. In Fig. 3b we compare our findings of the tuning capability with reports in the literature. Our observation of about 50% frequency tuning (marked by an orange circle) lies in the top range among the silicon-based MEMS/NEMS on-chip regulation devices. More importantly, the tuning capability is achieved by an operable voltage (2.9 V) for a typical microelectronic device.

Moreover, the vibrational pattern of the MGS resonator can be tuned notably by utilizing the ETM effect when buckling sets in. Fig. 3d shows the comparison between the vibrational patterns of the (1,1) mode captured before (*V*_DC_ = 0 V, left panel) and after (*V*_DC_ = 2.75 V, right panel) the buckling transition for the phase *ϕ* = 90° recorded by stroboscopic illumination. The spatial deflection of the vibrational motion under *V*_DC_ = 0 V (left panel) shows a usual sinusoidal envelope of the amplitude distribution, indicating a negligible asymmetry of the system. In contrast, the envelope of the amplitude distribution deviates from the sinusoidal shape at *V*_DC_ = 2.75 V, as shown in the right panel. The area with the maximal vibration amplitude shifts from the center to the area of lead 1, demonstrating a controlled symmetry breaking. This significant symmetry breaking can also be explained by the ETM effect: the heating above the buckling transition changes the internal stress distribution inhomogeneously resulting in an asymmetric stress distribution in the MGS device^33–35^.

### On-surface actuation of a MGS resonator

**Fig. 4:**
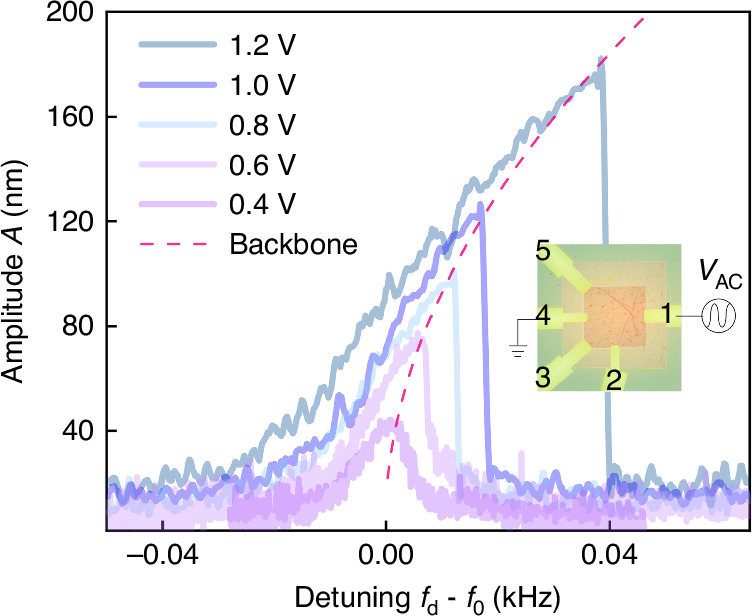
Nonlinear dynamic response curves of the MGS resonator actuated by the ETM effects with different input voltages *V*_AC_ ranging from 0.4 V to 1.2 V and measured by IWLI. The backbone trace is plotted as a red dashed line. The inset shows the circuit for the ETM drive, where no piezo is applied

Furthermore, the ETM effect also provides an easy way to realize on chip driving of resonances, corresponding to the scheme in Fig. 1d. When the AC voltage is applied to the metal leads of the MGS structure, the interface between G and metal starts to generate heat (mainly considered here) alternately. It results in a global increase of the temperature superimposed with a small modulation. Based on the ETM effect, such periodic temperature variation leads to alternating local stress (the ETM induced periodic force) as actuation (drive) force to the NEMS/MEMS device, similar to the photo-thermo-mechanical excitation by using an AC modulated laser^36^. The resonator can be driven by applying an AC voltage *V*_AC_ between leads 1 and 4, without any external (piezo) drive as shown in the inset of Fig. 4, where a series of resonance curves (*V*_AC_ from 0.4 V to 1.2 V) is plotted as a function of the detuning. We note that for the actuation, we inject *V*_AC_ at a frequency close to the *f*_0_ of the MGS resonator. In a perfectly symmetric device, the ETM effect relying on heating should give rise to an actuation at twice *f*_*d*_. Any lateral symmetry breaking will result also in responses at *f*_*d*_ itself. Hence, the ratio between the *f*_*d*_ and 2*f*_*d*_ response can be tuned by the geometry of the electrodes or the interface resistance. Here, the *I*-*V* curves in Supplementary Fig. S6 in the SI^27^ show a slight nonlinear relationship, typical for Schottky contacts. This results in different voltage drops across one contact area under positive and negative applied voltages, causing asymmetric power loading. Consequently, the period of temperature change on one electrode matches that of *V*_AC_, leading to a pronounced component at *f*_*d*_ instead of 2*f*_*d*_.

At *V*_AC_ = 0.4 V, the *Q* factor of the resonance is 2.3 × 10^4^, which is similar to the one obtained from the piezo-driven response in the linear regime at *V*_DC_ = 0 V in Fig. 3b. Increasing *V*_AC_ drives the system into the nonlinear regime, resulting in a slight shift of *f*_0_ of the (1,1) mode under different *V*_AC_. The Duffing nonlinearity of the resonator is determined to be *γ* = 2.5 × 10^22^ m^−2^s^−2^ when *V*_AC_ is smaller than 1.0 V.

When *V*_AC_ increases to 1.2 V, the nonlinearity increases due to the ETM-induced strain of the resonator. The ETM-induced strain modifies the potential symmetry, leading to the appearance of an asymmetric term in the mode’s potential energy^34^. This results in a cubic nonlinearity of the potential, i.e., a quadratic term in the equation of motion. The dynamics with broken symmetry at *V*_AC_ = 1.2 V can be well described by the potential:

3 $$V(q)=\frac{2\pi {f}_{0}^{2}}{2}{q}^{2}+\frac{{\gamma }_{3}}{3}{q}^{3}+\frac{{\gamma }_{4}}{4}{q}^{4}.$$

Here, *q* represents the deflection amplitude, and the potential includes a cubic nonlinearity (*γ*_3_) and a quartic (Duffing) nonlinearity (*γ*_4_). The parameter *γ*_3_ is related to the symmetry breaking. By solving the corresponding equation of motion, we extracted *γ*_3_ = 9.65 × 10^17^ m^−1^s^−2^ and *γ*_4_ = 5.38 × 10^23^ m^−2^s^−2^. The value of *γ*_4_ is one order of magnitude larger than the Duffing nonlinearity (*γ*) extracted previously, meaning that symmetry breaking significantly influences the nonlinearity. As a result, the presented MGS structure utilizing ETM effects provides new insights into controllable symmetry breaking through on-surface structures for silicon-based dielectric membrane resonators. Furthermore, the engineered tunable symmetry breaking enables the MGS structure to be highly versatile, thereby enabling numerous applications, some examples of which are sketched in the SI^27^.

Furthermore, we wish to comment on the robustness and versatility of our method for resonators. Previous methods for tuning the eigenfrequency, nonlinearity, vibrational pattern, and symmetry breaking require separate structures, such as a bottom and/or side gate^33,34^, an attached piezo actuation system^10^, or separate laser illumination^37^. In contrast, our MGS structure provides an on-surface integration possibility with an extremely large capability for tuning and driving. The ETM method relies solely on the parameters *R*, *f*_0_, *Γ*, *P* (*V*_DC_ or *V*_AC_), geometry, and material properties. All of these parameters can be directly extracted from experimental characterization or from a one-time fitting of the driven modes, making our method much simple and straightforward. Additionally, the ETM method provides a direct way to control the dynamic behavior under different symmetry conditions. Finally, we note that the dynamic response reported here has been recorded at the same frequency as the drive frequency *f*_*d*_ and not at its harmonic 2*f*_*d*_, as one could expect due the heat-related mechanism. We argue that differences of the M-G contacts result in a different heat dissipation for the two current directions which gives therefore rise to 1*f*_*d*_ periodicity of the excitation. We believe that there will be also a 2*f*_*d*_ component that, however, we cannot capture with our IWLI.

## Conclusion

In this study, we proposed a scheme for strong enhancements of tunability and functionality of silicon based device with graphene integration. We designed and fabricated a MEMS/NEMS device composed of a MGS structure bearing the potential for wide-range tuning and actuation based on ETM effects. We clarified the corresponding mechanisms and models for the MGS structure under different working scenarios. Firstly, by applying a DC voltage to the MGS structure, we can control the static deformation of the MEMS/NEMS device by utilizing the ETM effect, and the corresponding bilayer model is well established. This provides a direct way of motion and actuation control, especially for 2D-MEMS/NEMS devices. Secondly, the MGS structure achieves a large regulation capability on the dynamic properties of MEMS/NEMS resonator. The eigenfrequency is tuned over an extremely wide range (over 50%), a significant controllable symmetry breaking is introduced into the vibration system and the nonlinearity can be altered. Thirdly, by applying an AC voltage, on-surface driving of the membrane resonator can be achieved without external excitation. The AC modulated ETM effect excites the vibration of the resonator, even into the nonlinear regime. Finally, also the nonlinear properties can be significantly tuned by the symmetry breaking, and are quantitatively described by the Duffing nonlinear model extended by an additional cubic nonlinearity of the potential. The developed MGS structure opens up possibilities for MEMS/NEMS actuation applications and breaks the tuning limit of MEMS/NEMS devices, also for advanced applications of 2D nonlinear resonators^38,39^. Examples include controlled mode coupling/decoupling^37^, effective nonlinearity cancellation^34^, and energy conservation for the amplitude stabilization in persistent response without working in the nonlinear parametric coupling regime^40^. The underlying working principle is not unique to the material combination chosen here, i.e., to any other membrane-electrode material combination with sufficiently distinct thermal expansion coefficients. In the same spirit, also graphene could, in principle, be replaced by another sufficiently conductive thin material that provides a sizable interface resistance to the metal electrodes can be chosen^41,42^. Regarding the detection of the motion, the presented device concept can be completed by integrating also an on-chip electrical detection concept^10^ that enables the development of a fully-integrated on-chip actuation and detection system that can be miniaturized further and scaled up.

## Methods

### Sample fabrication

The SiN membrane is produced by wet-etching a 0.5-mm thick commercial (110) silicon wafer coated with a ~110-nm thick layer of LPCVD SiN on both sides in aqueous KOH. The sample presented here has a lateral size of 495 μm × 512 μm, and a thickness of 110 nm. The monolayer G is grown by chemical vapor deposition and transferred to the surface of the SiN membrane using a wet method^43^, covering the entire SiN membrane as well as part of the Si frame. The G is then patterned by using electron beam lithography to define the channel and leads, followed by an oxygen plasma etching to remove the unwanted parts of G. Next, the lead areas of G are contacted with Ti/Au top electrodes (total thickness of about 50 nm, marked from 1 to 5 in Fig. 1e) patterned by electron beam lithography and deposited by electron beam evaporation).

### Measurement scheme

The chip carrying the SiN sample is fixed to a piezo ring. The sample is placed in a vacuum chamber at room temperature with a base pressure *p* ≃ 1 × 10^−2^ mbar.

The surface of the membrane is characterized using IWLI with various light sources, as described in detail in ref.^44^. Dynamic deflections of the membrane can be excited by applying an AC voltage ${V}_{{\rm{exc}}}\cdot \sin (2\pi {f}_{d}t)$ to the piezo, resulting in an inertial excitation of the membrane. The excitation voltage is applied using a sinusoidal function generator, the phase of which can be synchronized with the stroboscopic light of the interferometer. In the study of the dynamic properties of MGS structure continuous light was used to record the resonance curves^45^, while stroboscopic light was used to measure the vibrational patterns^28^. Examples of different mode shapes are presented in the SI^27^. The mechanical properties (Young’s modulus and residual stress) are determined from the dispersion relation as described in refs. ^28,46^.

The two-point current-voltage (*I* − *V*) curves are slightly nonlinear, which suggests that the contact resistances are non-Ohmic. The two-point resistance of the G-M structure decreases from several tens of k*Ω* to around 10 k*Ω* as *V*_DC_ is increased from 0 to 3 V, depending on the lead combination, see the SI^27^. The resistance of the G channel is determined by the van-der-Pauw measurement^47^ and amounts to *R* = 1.65 k*Ω* for the present sample, see the SI^27^. Further details about the sample fabrication, setup, and fitting processes can be found in our previous works^28,45,46^.

## Supplementary information

Supplemental Material File #1
