# Supplementary material for: Silicon-based MEMS/NEMS empowered by graphene: a scheme for large tunability and functionality
Source: Microsyst Nanoeng. 2025 Jun 9;11:116. doi: 10.1038/s41378-025-00960-0 (PMC12149320; doi:10.1038/s41378-025-00960-0)
Supplement: Supplementary file 1 — Supplemental Material File #1 [file 41378_2025_960_MOESM1_ESM.pdf]

Supporting Information for: Silicon-based MEMS/NEMS Empowered by Graphene: A Scheme for Large Tunability and Functionality

Mengqi Fu,[1] Zhan Shi,[2] Bojan Bošnjak,[3] Robert H. Blick,[3] Elke Scheer,[1] and Fan Yang[1,4,*]

[1] *Fachbereich Physik, Universität Konstanz, 78457 Konstanz, Germany*
[2] *Department of Mechanics, Key Laboratory of Soft Machines and Smart devices of Zhejiang Province, Zhejiang University, 310058 Hangzhou, China*
[3] *Center for Hybrid Nanostructures, Universität Hamburg, 22761 Hamburg, Germany*
[4] *Dynamic Precision Micro&Nano Sensing Technology Research Institute, Chongqing, 400030 Chongqing, China*

Sample fabrication and setup

Sample fabrication

The SiN membrane is produced by wet-etching a 0.5 mm thick commercial (100) silicon wafer coated with a $\sim$ 110 nm thick layer of LPCVD SiN on both sides in aqueous KOH. The sample presented here has a lateral size of 495 μm $\times$ 512 μm, and a thickness of 110 nm. The monolayer Graphene (G) is grown by chemical vapor deposition and transferred to the surface of the SiN membrane using a wet method [S1], covering the entire SiN membrane as well as part of the Si frame after the wet transfer.

To pattern the G to include both the G channel and the contact area with metal leads, a layer of negative resist is spin-coated onto the surface of the G and patterned using electron beam lithography. An oxygen plasma etching process is then performed to remove the unwanted parts of the G. The area of the G after etching, metal leads, membrane, and the frame are labeled in Fig. S1. The designed overlap between the metal leads and the G ensures more even heating of the metal leads. After removing the negative resist in hot acetone, the metal lead area is patterned by a standard electron beam lithography process using a positive-resist system (one MMA-MAA copolymer layer and one PMMA layer). A layer of Ti/Au (thicknesses around 25 nm each) metal film is evaporated by electron beam evaporation followed by a lift-off process. The metal leads are marked from 1 to 5 in Fig. S1 as well as in Fig. 1(e) in the main text. Further details about the sample fabrication, setup, and fitting processes can be found in our previous works [S2–S4].

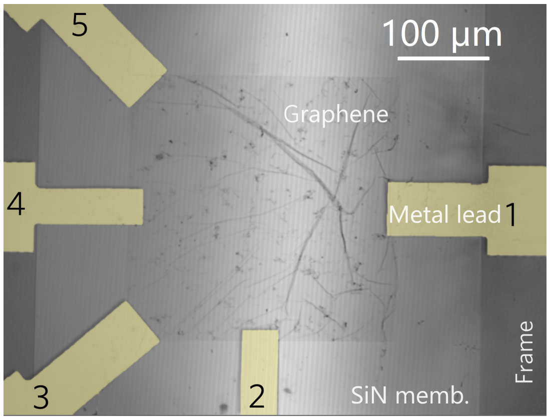

FIG. S1. Image of the MGS device captured by IWLI. The metal leads are marked from 1 to 5.

Measurement scheme

The sample with SiN membrane and G devices is glued to a printed circuit board (PCB) which has connections to external electronics. The metal leads are bonded to the PCB, so that an electric signal can be applied to them. The PCB is glued to a piezo ring that can vibrate perpendicular to the surface of the Si substrate of the sample under an AC voltage. Note that this piezo ring is only used in the measurements of the eigenfrequency and the nonlinearity of

the SiN membrane under V_{DC} in this work. The sample is placed in a vacuum chamber at room temperature. The chamber is connected to a pump which keeps the $p = 0.001$ mbar for the experiments. For $p \leq 0.01$ mbar, the damping is dominated by intrinsic damping mechanisms of the membrane and its clamping, and losses due to coupling to the atmosphere are negligible [S2]. The surface of the membrane is examined using IWLI with various light sources, as described in detail in [S5].

As an illustration, Fig. S2 (a) displays the captured vibrational motion of the ground mode (labeled as (1,1) mode) of a G-covered SiN membrane resonator at a phase $\sim 90°$ using stroboscopic light with phase locking of the IWLI. A drumhead vibration deflection can be observed in the center of the membrane.

Mechanical properties: Eigenfrequencies, vibrational patterns, dispersion relation, and nonlinearity

The eigenfrequencies of the flexural modes of a rectangular membrane can be calculated using the formula:

$$f_{m,n} = \sqrt{(\sigma_{xx} m^2/L_{\rm w}^2 + \sigma_{yy} n^2/L_{\rm h}^2)/(4\rho)}\,, \tag{S1}$$

here the integers m and n indicate the number of deflection maxima in the two spatial directions of the membrane plane, $L_{\rm w}$ and $L_{\rm h}$ represent the lateral size of the membrane along the x and y direction, respectively. σ_{xx} and σ_{yy} are the residual stress in x and y direction and ρ is the density of the material.

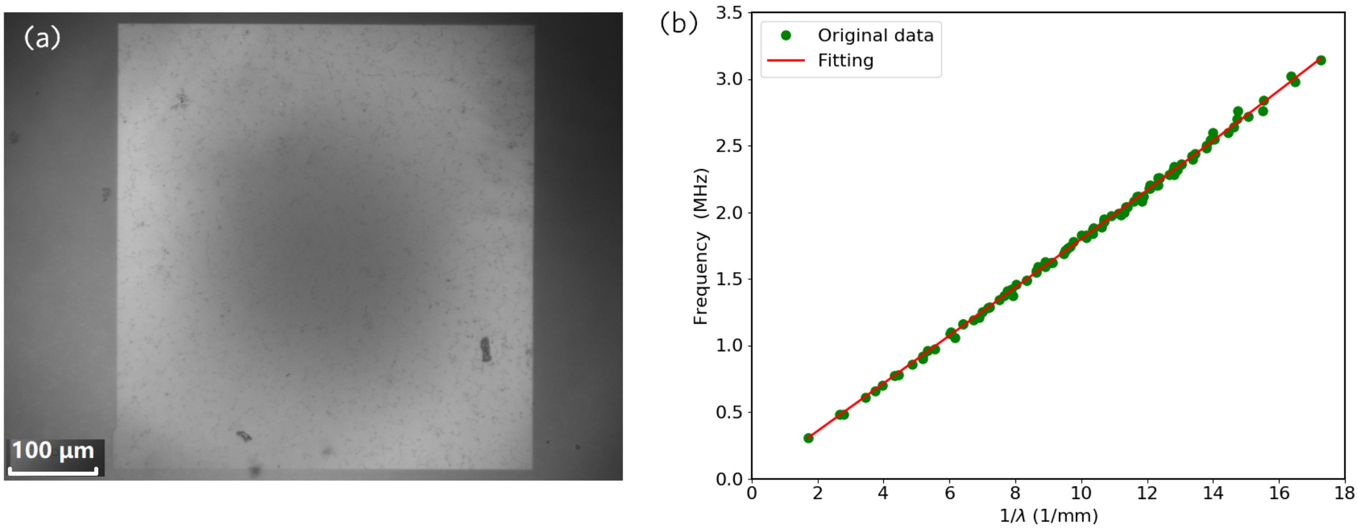

FIG. S2. (a) Camera view of the (1,1) mode vibration motion of a G+SiN membrane from the same batch as the sample shown in main text. The motion is captured by stroboscopic light with phase locking during IWLI before the addition of the M leads. (b) By capturing the vibrational motion of different modes, we construct the dispersion relation of the G+SiN membrane before patterning the metal leads. The eigenfrequencies of the different modes (blue dots) are plotted corresponding to their wave number. The dispersion relation is fitted by Eq. S2 and plotted as red solid line.

Figure S3 (a) - (f) give examples of measured deflection amplitude profiles, which display the vibration amplitude image of the (1,1) mode (ground mode), (1,2) mode, (2,1) mode, (2,2) mode, (2,3) mode, and (3,2) mode, respectively. The dispersion relations are obtained from time series of these images as described in [S3]. Young's modulus and the residual stress can be determined from the dispersion relation Eq. (S2):

$$\omega = \sqrt{\frac{Eh^2}{12\rho\,(1-\nu^2)}k^2 + \frac{\sigma_{xx}}{\rho}k}. \tag{S2}$$

Here h, ρ, ν and E are the thickness, density, Poisson's ratio and Young's modulus of the membrane, respectively. The stress in the x-direction of propagation is σ_{xx}. The extracted values for the frequency f and the wave number $1/\lambda$ data from $\vec{k}$-space fit method are used to fit the curve. $\omega = 2\pi f$ and $k = 2\pi\frac{1}{\lambda}$ are defined. The thickness, density and Poisson's ratio are adapted as $h = 110\,\mathrm{nm}$, $\rho = 3180\,\mathrm{kg/m^3}$ and $\nu = 0.27$, respectively, in the present case. The

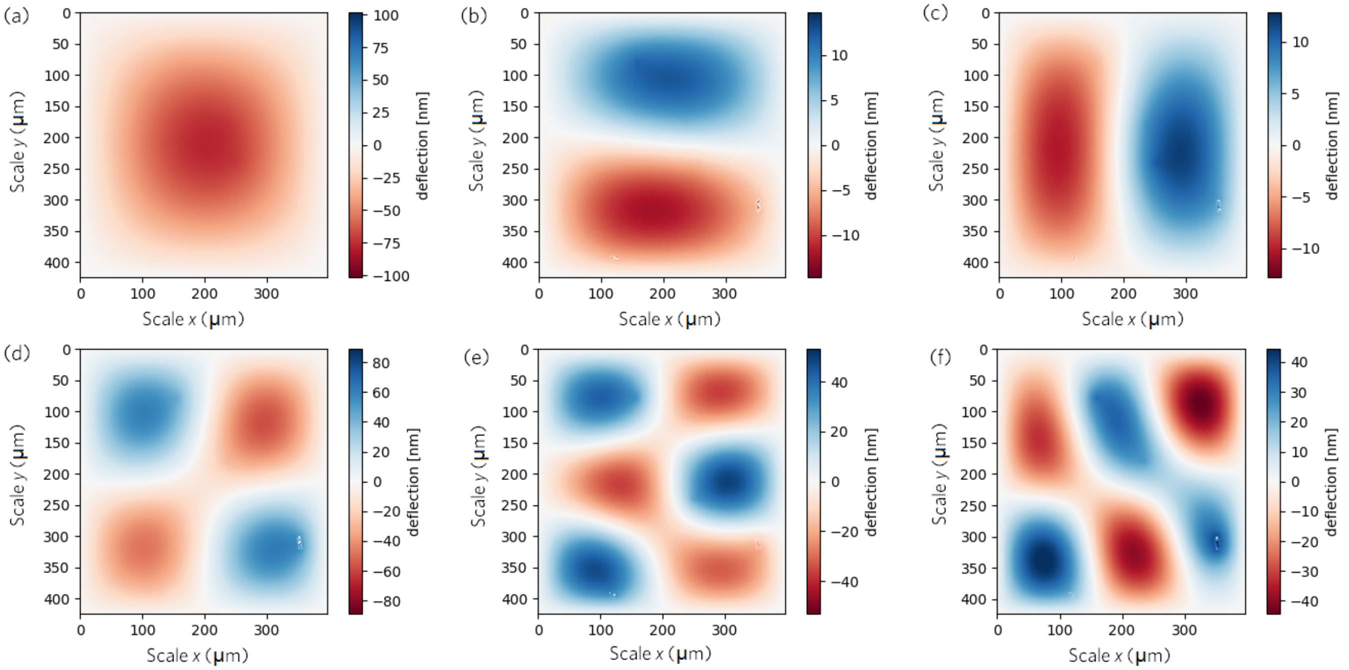

FIG. S3. Mode shapes of several representative experimentally captured vibration modes of a G+SiN membrane with slightly smaller lateral dimension. (a) (1,1) mode at $\sim 260\,\text{kHz}$. (b) (1,2) mode at $\sim 460\,\text{kHz}$. (c) (2,1) mode at $\sim 490\,\text{kHz}$. (d) (2,2) mode at $\sim 525\,\text{kHz}$. (e) (2,3) mode at $\sim 760\,\text{kHz}$. (f) (3,2) mode at $\sim 780\,\text{kHz}$.

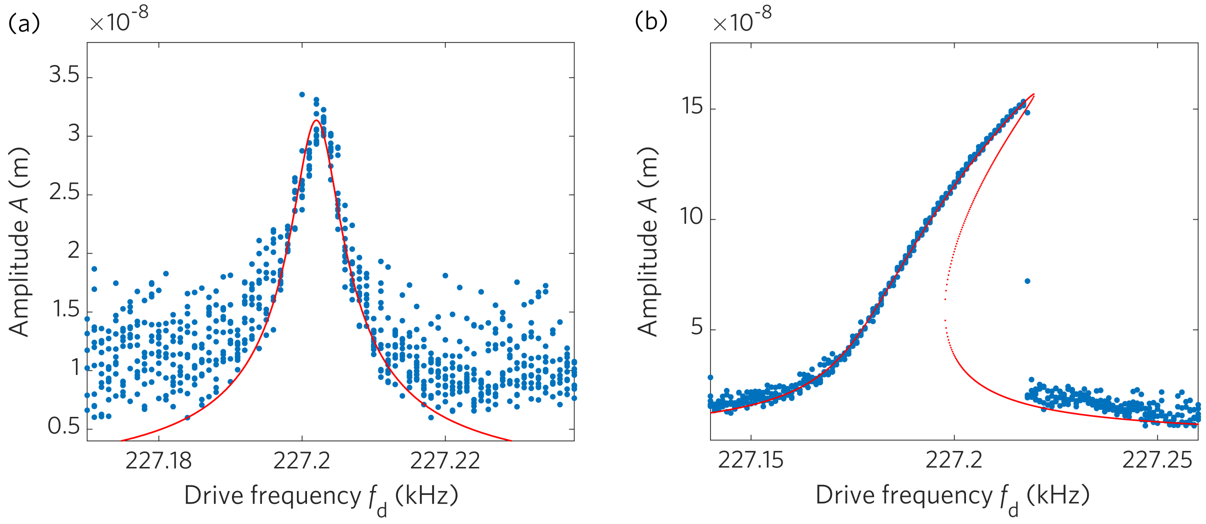

FIG. S4. Linear and nonlinear amplitude response of the MGS resonator driven with (a) $V_{\text{exc}} = 1.8\,\text{mV}$ and (b) $V_{\text{exc}} = 9.1\,\text{mV}$, respectively. The measured amplitudes (captured by IWLI) are plotted as blue dots, and the theoretical calculation of the Duffing model is plotted as red line.

estimated Young's modulus and stress are 213 GPa and 0.101 GPa, respectively. The Duffing model is commonly used to describe the nonlinear effects in nanomechanical membrane resonators with the equation of motion:

$$\ddot{q}(t) + 2\pi f_0^2 q(t) + 2\Gamma \dot{q}(t) + \gamma q^3(t) = F_{\text{d}} \cos(2\pi f_d t) \tag{S3}$$

where f_0,Γ, γ, and F_d are the eigenfrequency, damping, nonlinearity and the drive force, respectively. As an example, we analyze the nonlinear amplitude response of the MGS resonator driven with $V_{\text{exc}} = 9.1\,\text{mV}$. The same data as shown in the main text in Fig. 3 (b) is presented in Fig. S4. The theoretical modeling indicates that the MGS membrane resonator presents the following mechanical properties: $f_0 = 227.18\,\text{kHz}$, $2\Gamma = 8.0\,\text{Hz}$, $\gamma = 3.5 \times 10^{22}\ \text{m}^{-2}\text{s}^{-2}$, and F_d

= 9.8 N/kg.

The Duffing nonlinearity of the (1,1) mode can be extracted from the backbone trace characterized by the maximum amplitude and corresponding detuning at a given force, using

$$(f_{d,max} - f_0) = \frac{3\gamma}{8f_0} A_{max}^2 \,. \tag{S4}$$

Typical mechanical response curves of the MGS device from the linear to the nonlinear regime of the (1,1) mode are shown in Fig. 3 (b) in the main text. The extracted backbone trace shown as the red dashed line indicates the nonlinearity $\gamma = 3.64 \times 10^{22}$ $\mathrm{m^{-2}s^{-2}}$, consistent with the result from the fitting of the Duffing curve.

f_0 can be calculated by Eq. (S1) with the help of the residual stress determined from the dispersion relation. Moreover, the γ can be calculated by the geometric nonlinearity [S6, S7]:

$$\gamma = \frac{3\pi^4}{16\rho}\left(\frac{E_{xx}m^4}{L_\mathrm{w}^4} + \frac{E_{yy}n^4}{L_\mathrm{h}^4}\right), \tag{S5}$$

the relation $E_{xx} = E_{yy} = E$ is used considering an isotropic system, and the nonlinearity can be calculated as $\gamma = 3.8 \times 10^{22}$ $\mathrm{m^{-2}s^{-2}}$, which is similar to the experimental result.

Sheet resistance and contact resistance of G

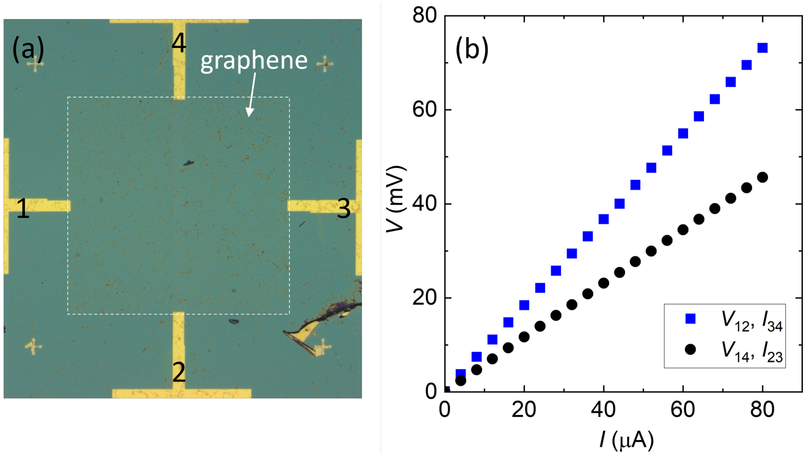

FIG. S5. (a) Optical image of the device used in Van-der-Pauw measurement. (b) I-V measured from two configurations of Van-der-Pauw measurement.

The total device resistance obtained from the two-point measurement includes the resistance of the G channel and the contact resistances between the M leads and G. To determine their contribution separately, we measured the sheet resistance ($R_\square$) of G by the Van-der-Pauw method [S8] on another MGS sample, as shown in Fig. S5. The G layer of the device was grown by the same method, and the fabrication processes of the device are similar with the MGS device investigated in the manuscript. The $R_\square$ is calculated to be around 1.65 kΩ which is similar to reported values [S9]. By using this value and the fact that the G channel is square shaped, we can estimate the channel resistance of G between 1 and 4 to be around 1.65 kΩ. As shown in the two-point measurements between lead 1 to 2, 3, 4, and 5, respectively, in Fig. S6, the two-point resistance of the G-M structure decreases from several tens of kΩ to around 10 kΩ as V_DC is increased from 0 to 3 V, which is much larger than the resistance of the G channel. Hence, the contacts between G and M provide the dominating contribution to the total device resistance and thus the heating effects.

In Fig. S7 we show the resonance curves measured under different V_DC between electrodes 1 and 4 and for excitation voltage $V_\mathrm{exc} = 9.1$ mV. By fitting the Duffing model to the experimental data the eigenfrequency was determined and found to shift to smaller values, in agreeement with the data obtained for smaller excitation in the linear regime, shown in Fig. 3 of the main text.

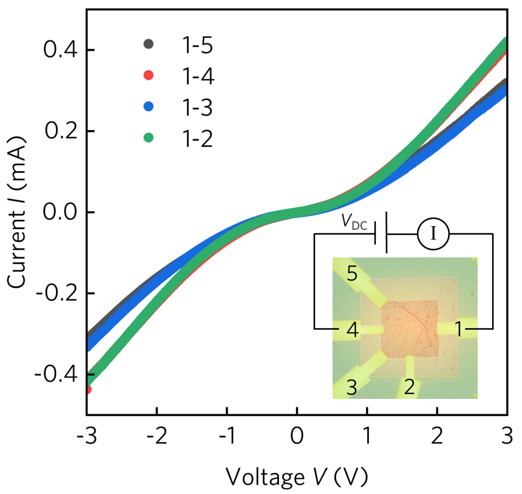

FIG. S6. Electric properties of the G. (a) I-V curves between different leads on the membrane surface. Inset: Microscope image of the MGS device with measurement circuit scheme. The metal leads are labeled from 1 to 5; the SiN membrane holds the G and metal structures and is clamped by a Si frame.

Thermomechanically controlled symmetry breaking: static deformation and vibration patterns

The spatial deflection of the membrane under different V_{DC} (2.65 V, 2.75 V, 2.85 V and 2.9 V) applied between leads 1 and 4 is captured by the IWLI and plotted together with line cuts through the center in Fig. S8 (a)-(d). The spatial deflection increases with V_{DC} due to the larger stress generated at the G-M interface.

The expansion of the M reduces the residual strain along the x-axis of the SiN membrane between leads 1 and 4 and also exerts a compressive stress on this area. The compressive stress is also oriented along the x-axis from the outer ends (on the frame) of the M leads to their inner ends (on the SiN membrane), squeezing the SiN membrane between leads 1 and 4. The SiN layer exhibits the most pronounced deflections around the inner ends of the M leads because of the relatively smaller rigidity of the thin SiN layer. When V_{DC} is larger than 2.75 V, also the center of the membrane develops corrugations. The pronounced deflection is not only observed when V_{DC} is applied to leads 1 and 4, but also other combinations of the leads show this effect, as shown in Fig. S10.

The ETM induced static deformation provides a possible way to monitor the symmetry breaking of the membrane without a capacitive 3D structure (e.g., a back gate located under the membrane) which simplifies the fabrication process. We also extract the strain of the most strongly deformed area and plot it in Fig. S9. The strain is extracted from the spatial deformation along the x-axis between of 370 μm and 450 μm (indicated in Fig. S9 (a) and (b)). The strain can be obtained by the following equation:

$$\mathrm{Strain} = \frac{L_{\mathrm{SiN},P} - L_{\mathrm{SiN}}}{L_{\mathrm{SiN}}}, \tag{S6}$$

here $L_{\mathrm{SiN},P}$ represents the integrated length in the range of the surface between 370 - 450 μm under different heating power P generated by V_{DC}, L_{SiN} represents the original length between 370 - 450 μm on the surface (i.e., 80 μm) with $P = 0$. In Fig. S9 (c), the extracted strain values with increasing P are plotted as blue dots. A 5th order polynomial function can describe the relation between the ETM-induced strain and the input power, plotted as the red dashed line.

Here we characterize the spatial deflection of the (1,1) mode with and without symmetry breaking, by stroboscopic IWLI measurements. We applied $V_{\mathrm{DC}} = 0$ V and $V_{\mathrm{DC}} = 2.75$ V to leads 1 and 4 to demonstrate the ETM controlled symmetry breaking. The device is driven by the piezo with $V_{\mathrm{exc}} = 0.5$ V. The drive frequency is swept up from below to slightly above the respective eigenfrequency (the eigenfrequency is shifted to smaller values due to the heating effects as well as by the symmetry breaking introduced by V_{DC}). When $V_{\mathrm{DC}} = 0$ V, there is no overall heating and symmetry breaking in the system. The spatial deflection of the vibrational motion of the (1,1) mode shows a usual sinusoidal envelope of the amplitude distribution, which is symmetric with respect to the x and y axis. The two patterns are captured at the phase of $\phi = 90°$ and 270° by stroboscopic measurement, shown in Fig. S11 (a) and (b), respectively. An overall heating and symmetry breaking is observed by applying $V_{\mathrm{DC}} = 2.75$ V to the leads 1 and

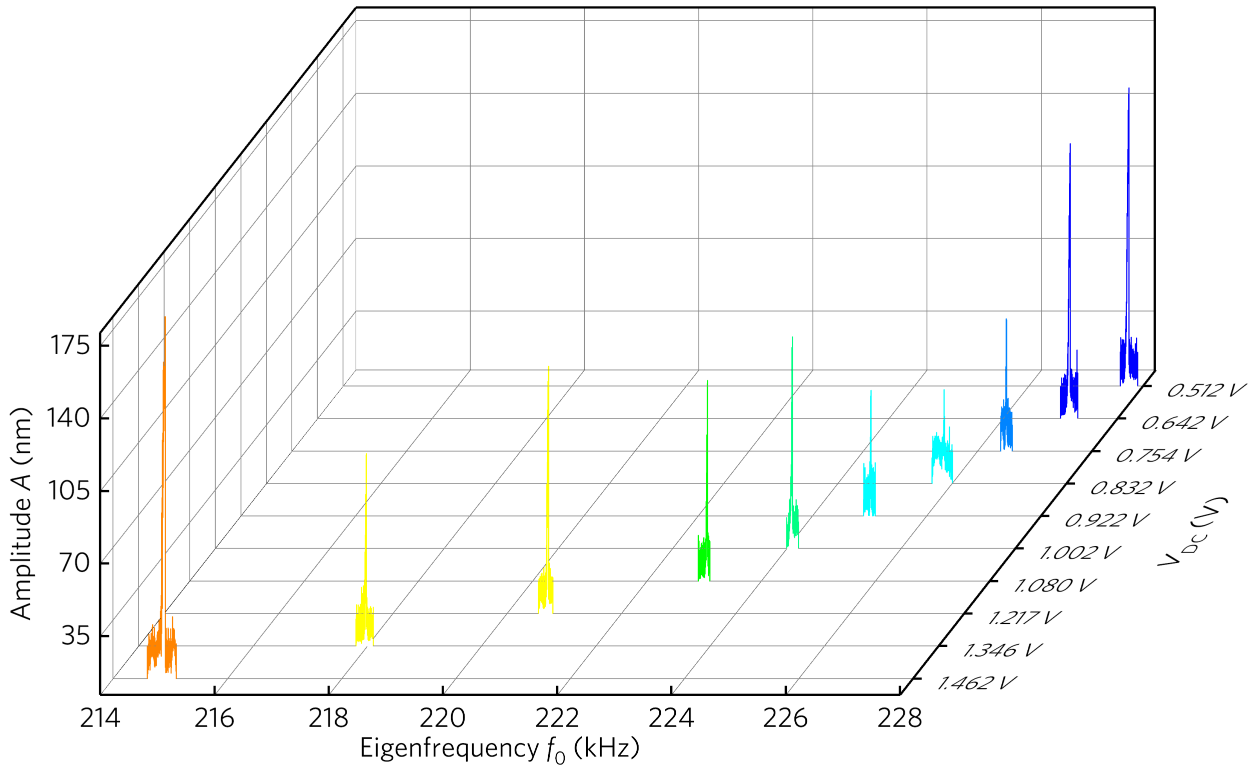

FIG. S7. Resonance curves in the Duffing regime measured by IWLI under different V_{DC}. The observed eigenfrequency shift is roughly proportional to the power P.

4. As shown in Fig. S8 (b), the membrane shows a static deformation with maximal height $\approx 500\,\mathrm{nm}$ (representing symmetry breaking). A voltage V_{exc} of the same size is applied to the piezo and the frequency is swept around f_0 which is significantly tuned by the overall heating and symmetry breaking. The patterns are captured at $\phi = 90°$ and $270°$ by stroboscopic measurement as well and are shown in Fig. S11 (c) and (d). Significant symmetry breaking in the dynamic regime can be observed. The maximum deflection amplitude is no longer in the center of the membrane but shifted to the side of lead 1, where the most pronounced static deformation was obtained by V_{DC}.

Model for curvature radius: modified Timoshenko model

We built our model based on certain simplifications motivated by the geometry and properties of our experimental device:
(1) The bending of the MGS structure is subject to a uniform heating from T_0 to T;
(2) The metal lead is considered as one continuous layer and the expansion effect of G is neglected because of its ultrasmall thickness;
(3) The MGS structure is considered as a cantilever with prestress (equal to the residual stress of the SiN membrane) applied on the free end of SiN;
(4) The difference in the thermal expansion coefficients remains constant during heating such that the friction at the supports can be neglected.

If the thermal expansion coefficient of the metal layer and SiN, α_{M} and α_{SiN}, are different, the heating will produce bending of the MGS structure similar to the Timoshenko bilayer cantilever [S10]. E_{M} and E_{SiN} denote their Young's modulus, h_{M} and h_{SiN} their thicknesses, $h = h_{\mathrm{M}} + h_{\mathrm{SiN}}$ the total thickness. The width of the cantilever is taken as equal to unity.

The following analysis is made on the assumption that cross sections along the x-axis of the M leads originally were planar. The cross section perpendicular to the x-axis remains constant during the bending, i.e. the curvature in the

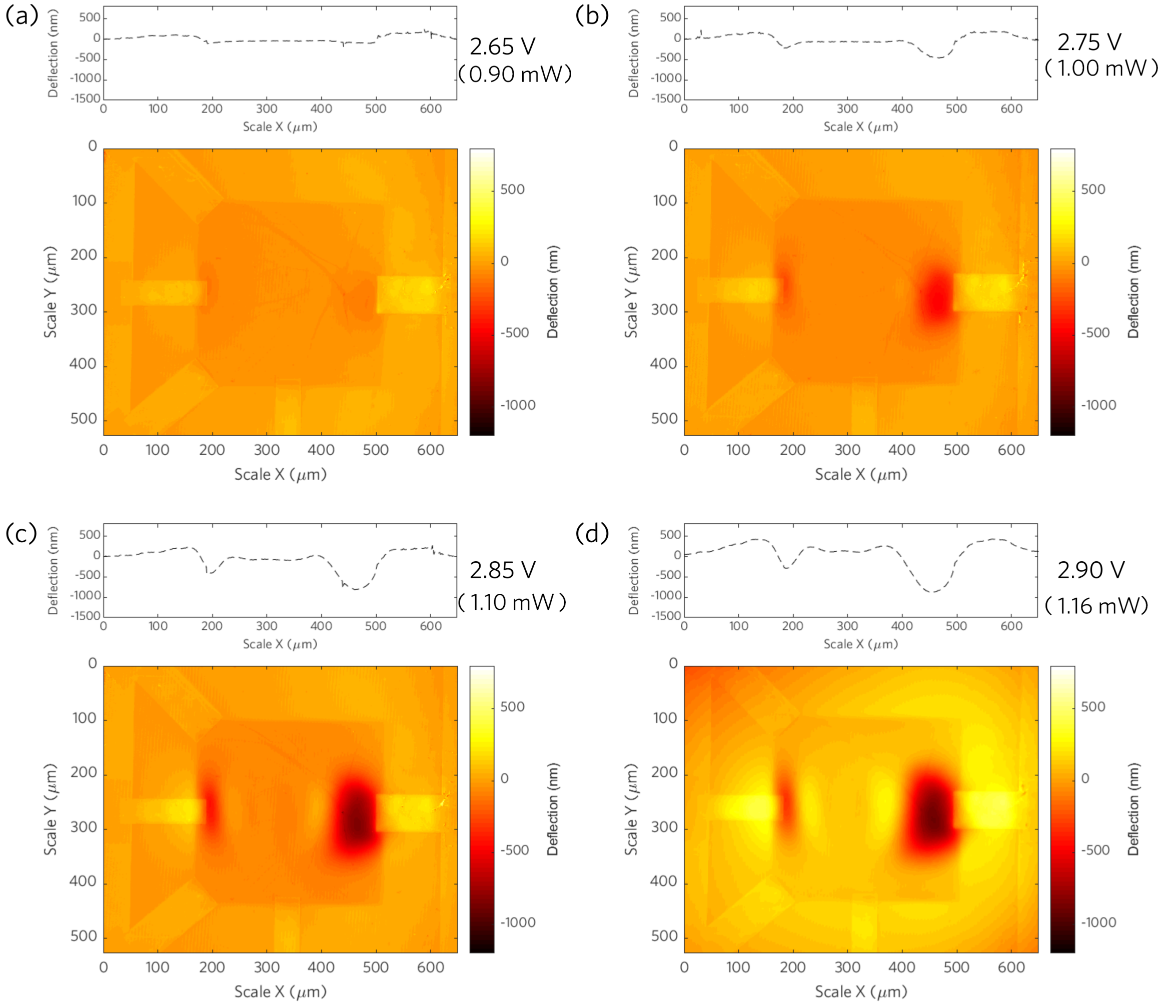

FIG. S8. Optical-profilometry characterization of voltage-induced thermal static deformation of the resonator. A line cut of the image at $y = 290$ μm is plotted in the upper panel. (a) $V_{\mathrm{DC}} = 2.65\,\mathrm{V}$. (b) $V_{\mathrm{DC}} = 2.75\,\mathrm{V}$. (c) $V_{\mathrm{DC}} = 2.85\,\mathrm{V}$. (d) $V_{\mathrm{DC}} = 2.90\,\mathrm{V}$.

y-axis is negligible due to the small width of the M leads.

Since $\alpha_{\mathrm{M}} > \alpha_{\mathrm{SiN}}$, the M leads elongate more than SiN and therefore the deflection is convex upwards [S10]. For the metal on the convex side, all forces acting on the cross section can be represented by an axial compressing force N_{M} and bending moment B_{M}. The forces acting over the section of SiN on the concave side can be represented by an axial tensile force N_{SiN}, a residual stress $N_{\mathrm{res}} = h_{\mathrm{SiN}}\sigma_{\mathrm{SiN}}$, and bending moment B_{SiN}. Since it is assumed that no other external forces except residual stress are acting on the cantilever, all forces acting over any cross section of the cantilever must compensate each other. Hence we obtain:

$$N_{\mathrm{M}} = N_{\mathrm{SiN}} + N_{\mathrm{res}} = N \tag{S7}$$

and

$$\frac{N_h}{2} = B_{\mathrm{M}} + B_{\mathrm{SiN}}. \tag{S8}$$

Letting r_c represent the curvature radius of the cantilever, $E_{\mathrm{M}}I_{\mathrm{M}}$ is the flexural rigidity of the metal and $E_{\mathrm{SiN}}I_{\mathrm{SiN}}$ represents the flexural rigidity of the SiN. We define $B_{\mathrm{M}} = \frac{E_{\mathrm{M}}I_{\mathrm{M}}}{r_c}$ and $B_{\mathrm{SiN}} = \frac{E_{\mathrm{SiN}}I_{\mathrm{SiN}}}{r_c}$, combining with Eq. (S8) we get:

$$\frac{N_h}{2} = \frac{E_{\mathrm{M}}I_{\mathrm{M}} + E_{\mathrm{SiN}}I_{\mathrm{SiN}}}{r_c}. \tag{S9}$$

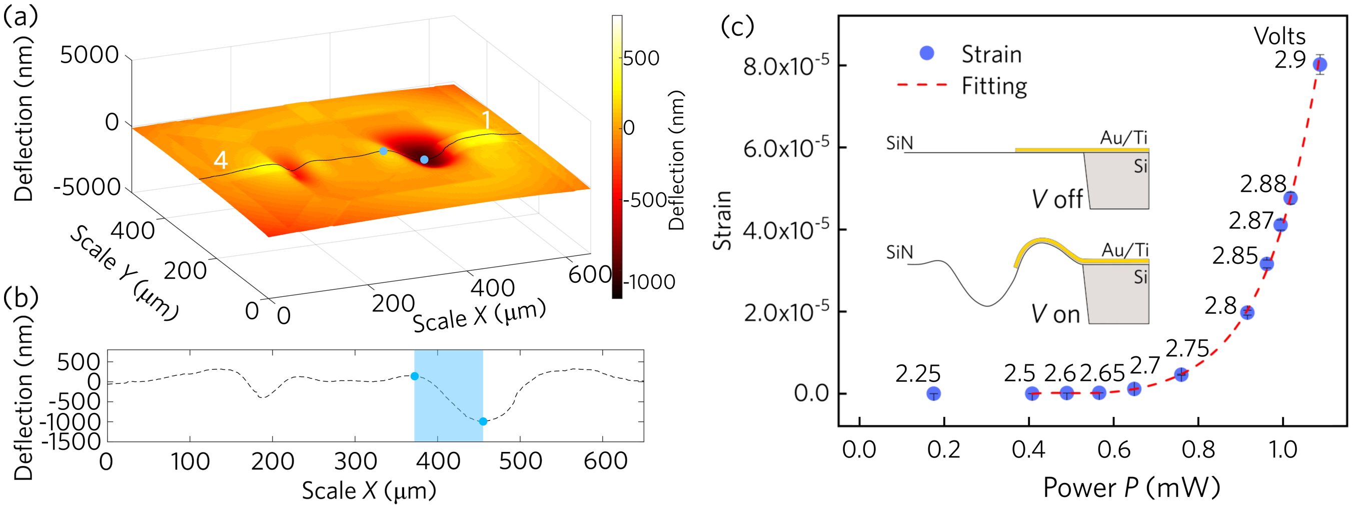

FIG. S9. Schemes demonstrating the controlled static deformation and the extracted strain of the SiN membrane.(a) IWLI image of the spatial deflection of the MGS resonator at $V_{\mathrm{DC}} = 2.9\,\mathrm{V}$. (b) Deflection profile of the resonator at the position on the membrane surface indicated by the black line in (a). The selected boundary ($370\,\mu$m, $450\,\mu$m) of the deflected SiN membrane in x-axis for strain calculation is marked as blue dots. (c) The strain is induced by the ETM effect of the MGS structures of lead 1 and 4. The strain is extracted from the spatial deflection along the x-axis between $370\,\mu$m and $450\,\mu$m. The current is converted from the input V_{DC} and the measured resistance. The red dashed line indicates a 5th order polynomial fitting.

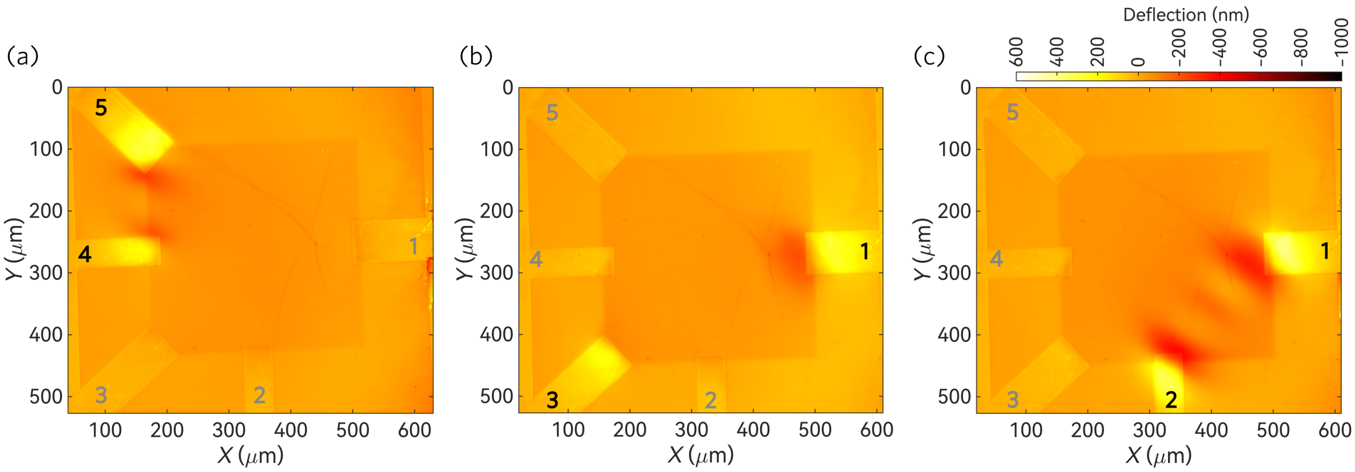

FIG. S10. Optical-profilometry characterization of voltage-induced thermal static deformation between different electrodes of the resonator. The labels of the electrodes to which the DC voltage $V_{\mathrm{DC}} = 2.75\,\mathrm{V}$ is applied are marked in black, the labels of the unused electrodes are marked in gray. (a) V_{DC} applied between electrodes 4 and 5. (b) V_{DC} applied between electrodes 1 and 3. (c) V_{DC} applied between electrodes 1 and 2. The deflections shown in (a) - (c) share the same color scale.

And by using Eq. (S7) and Eq. (S9), N_{M} and N_{SiN} adopt the following form:

$$N_{\mathrm{M}} = \frac{2(E_{\mathrm{M}} I_{\mathrm{M}} + E_{\mathrm{SiN}} I_{\mathrm{SiN}})}{r_c h}, \tag{S10}$$

$$N_{\mathrm{SiN}} = \frac{2(E_{\mathrm{M}} I_{\mathrm{M}} + E_{\mathrm{SiN}} I_{\mathrm{SiN}})}{r_c h} + N_{\mathrm{res}}. \tag{S11}$$

Another equation for calculating P and r_c can be obtained from the consideration of deformation. On the surface of M and SiN, the unit elongation occurring in the longitudinal direction must remain equal. This results in the

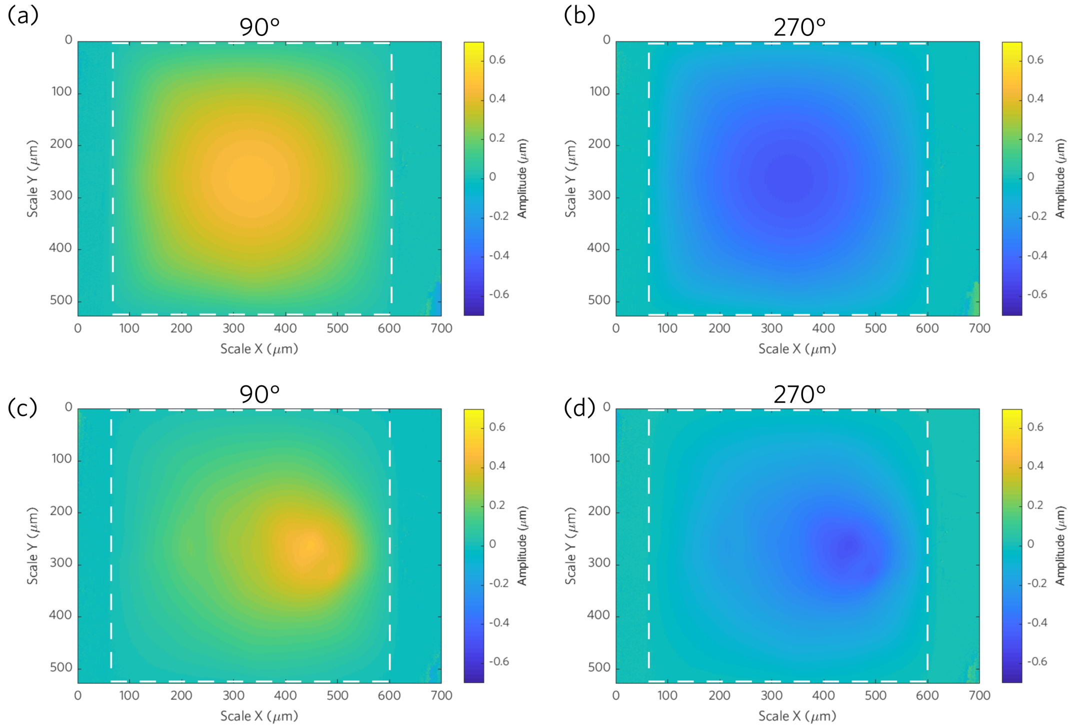

FIG. S11. Optical-profilometry characterization of the influence of the symmetry breaking onto the vibrational patterns of the (1,1) mode. (a) IWLI-captured vibration patterns without symmetry breaking ($V_{\rm DC} = 0\,$V, phase $\phi = 90°$. (b) Without symmetry breaking ($V_{\rm DC} = 0\,$V, $\phi = 270°$. (c) With symmetry breaking ($V_{\rm DC} = 2.75\,$V, $\phi = 90°$. (d) With symmetry breaking ($V_{\rm DC} = 2.75\,$V, $\phi = 270°$).

equation

$$\alpha_{\rm M}\left(T-T_0\right)-\frac{N_{\rm M}}{E_{\rm M}h_{\rm M}}-\frac{h_{\rm M}}{2r_c}=\alpha_{\rm SiN}\left(T-T_0\right)+\frac{N_{\rm SiN}}{E_{\rm SiN}h_{\rm SiN}}+\frac{h_{\rm SiN}}{2r_c} \tag{S12}$$

Using Eq. (S10) and Eq. (S11), Eq. (S12) becomes

$$\left(\alpha_{\rm M}-\alpha_{\rm SiN}\right)\left(T-T_0\right)-\frac{N_{\rm res}}{E_{\rm SiN}h_{\rm SiN}}=\frac{2\left(E_{\rm M}I_{\rm M}+E_{\rm SiN}I_{\rm SiN}\right)}{r_c h}\left(\frac{1}{E_{\rm M}h_{\rm M}}+\frac{1}{E_{\rm SiN}h_{\rm SiN}}\right)+\frac{\left(h_{\rm M}+h_{\rm SiN}\right)}{2r_c}, \tag{S13}$$

which leads to

$$\frac{1}{r_c}=\frac{\left(\alpha_{\rm M}-\alpha_{\rm SiN}\right)\left(T-T_0\right)-\frac{N_{\rm res}}{E_{\rm SiN}h_{\rm SiN}}}{\frac{2\left(E_{\rm M}I_{\rm M}+E_{\rm SiN}I_{\rm SiN}\right)}{h}\left(\frac{1}{E_{\rm M}h_{\rm M}}+\frac{1}{E_{\rm SiN}h_{\rm SiN}}\right)+\frac{\left(h_{\rm M}+h_{\rm SiN}\right)}{2}}. \tag{S14}$$

With the definitions $\frac{h_{\rm M}}{h_{\rm SiN}}=h'$, $\frac{E_{\rm M}}{E_{\rm SiN}}=E'$, here $I_{\rm M}=\frac{{h_{\rm M}}^3}{12}$ and $I_{\rm SiN}=\frac{{h_{\rm SiN}}^3}{12}$, the curvature of the MGS structure follows the general equation:

$$\frac{1}{r_c}=\frac{\left(6\left(\alpha_{\rm M}-\alpha_{\rm SiN}\right)\left(T-T_0\right)-\frac{\sigma_{\rm SiN}}{E_{\rm SiN}}\right)\left(1+h'\right)^2}{h\left(3\left(1+h'\right)^2+\left(1+h'E'\right)\left(h'^2+\frac{1}{h'E'}\right)\right)}. \tag{S15}$$

Here $T-T_0$ is the temperature rise of the metal electrode on the membrane.

Determination of the temperature increase $T - T_0$

The temperature at the heated part of the MGS structure at different $V_{\rm DC}$ is estimated by assuming a steady state between Joule heat load $P_{\rm in} = IV$ and heat dissipation $P_{\rm out}$ via heat conduction and radiation. Neglecting heat

TABLE S1. Sample properties

Domain	Dimensions (μm)	Thermal expansion coefficient
Graphene	$300 \times 300 \times 0.001$	$-3.2 \cdot 10^{-6}/K$
Electrode 1	$100 \times 60 \times 0.025$ (Ti) + $100 \times 60 \times 0.025$ (Au)	Au ($14.2 \cdot 10^{-6}/K$)
Electrode 2	$100 \times 40 \times 0.025$ (Ti) + $100 \times 40 \times 0.025$ (Au)	Ti ($7.6 \cdot 10^{-6}/K$)
SiN membrane	$495 \times 512 \times 0.11$	$3.2 \cdot 10^{-6}/K$

resistance between Au and G and G and SiN and radiation losses, the dissipation mainly takes place through heat conduction along the metal electrode to the Si frame, $\dot{Q}_{\rm M} \simeq \frac{\kappa_{\rm M} w_{\rm M} d_{\rm M}}{L_{\rm M}} \Delta T$ and laterally along the SiN membrane $\dot{Q}_{\rm SiN} \simeq \frac{\kappa_{\rm SiN} w_{\rm SiN} d_{\rm SiN}}{L_{\rm SiN}} \Delta T$. Using literature values for the thermal conductivity ($\kappa_{\rm SiN} = 10\,{\rm W/(m{\cdot}K)}$ and $\kappa_{\rm M} = 318\,{\rm W/(m{\cdot}K)}$) and a simplified onedimensial model and the electrode dimensions $L_{\rm M} = 110\,\mu$m, $W_{\rm M} = 60\,\mu$m, $h_{\rm M} = 30\,$nm, $d_{\rm SiN} = 110\,$nm we estimate that $\dot{Q}_{\rm SiN} \simeq 1.5 \cdot 10^{-7}\,$W/K is about two orders of magnitude smaller than $\dot{Q}_{\rm M} \simeq 4.6 \cdot 10^{-6}\,$W/K. These considerations justify the simple Fourier law Eq. (2) in the main text.

Considering the resistance contribution of the contact resistances, thermal radiation and heat conductance of the SiN layer perpendicular to the MGS, the heat exerted to the MGS structure is estimated to be roughly 85% of the total generated heat. The eigenfrequency shift $\delta f_0 \simeq 40\,$kHz can be estimated from Fig. 3 (a) in the main text with $V_{\rm DC} = 2.25\,$V, typically corresponding to a temperature increase of around 80 K ($\sim 500\,$Hz/K [S2]), corresponding to an average temperature of the device of $\sim$ 105 ℃, similar to the estimation (85 ℃) by Fourier's law.

To independently confirm the temperature gradient along the metal lead and the length L_M over which the temperature decays, we performed measurements of the temperature-induced Raman shift with the test sample shown in Fig. S12. The test sample is a MGS device as the one studied before. In addition, we attached two pieces of ultra-thin hexaboronnitride (hBN) to one of the metal leads, one on the part on top of the membrane (P1) and one on the part on the Si frame (P2). The Raman measurements were performed at the positions marked with the black circles. First we calibrated the temperature shift and wave-number relation and found $0.3\,{\rm cm}^{-1}/10\,$K and then the Raman peak of the hBN for different voltages applied to the MGS device leads. Note, that the absolute values of the power load required to obtain a particular temperature, cannot be directly compared, because the contact resistances are very different from the the one discussed in the main text. The results show that when the total power reaches 5.6 mW, the Raman shift of hBN is about $3\,{\rm cm}^{-1}$, corresponding to a temperature increase of the metal electrode is about 100 K. Next, we focused the laser to the hBN on the metal electrode on the frame, and found no shift of the Raman peak, confirming that temperature at point 2 remains at room temperature $\pm 5\,$K, given by the resolution of the Raman measurement. P1 and P2 are separated by the distance L_M, i.e,. is the length of the lead on the membrane. Finally we shifted the laser spot to a point P3 (not shown) a bit closer to the edge of the frame, we found a Raman peak shift of $0.3\,{\rm cm}^{-1}$ and hence a slight increase of about 10 K. This means that P2 is the position at which the temperature just decayed to room temperature.

COMSOL simulation of temperature distribution and deformation of the MGS

To testify the rationality of our simplifications in the analytic model and support our experimental results, the Finite-Elements-Analysis (FEA) software package COMSOL Multi physics (version 6.1, academic license from Eidgenössische Technische Hochschule Zürich (ETH Zürich)) is utilized to simulate the temperature distribution and deformation caused by the ETM effects on the MGS. The dimensions of the device under test are presented in Table S1, which matches the structure and dimensions of the MGS device shown in Fig. S1 and Fig. 1 in the main text. Since the quantum transport properties of a monolayer G cannot adequately be described by COMSOL, and in the present experiment we just use it as heater with a particular resistance, we mimicked it by a thin layer with thickness 1 nm with a resistivity adjusted such that it results in a resistance close to the experimental value. A prestress of 0.101 GPa (as measured in the experiment) is applied to the four edges of the SiN membrane. Details of the physics modules setup and corresponding domains can be found in Table S2.

A static voltage $V_{\rm DC} = 2.85\,$V is applied to the M electrodes. In order to approximate the relatively large contact resistance between G and M observed in our experiments, we enhanced the resistivity of both electrodes to $3.6{\times}10^{-8}$ Ωm. With these parameters we achieved a generated current of approximately 0.315 mA, which is close to the experimental value (around 0.34 mA). In Fig. S14, we present the temperature distribution and deformation

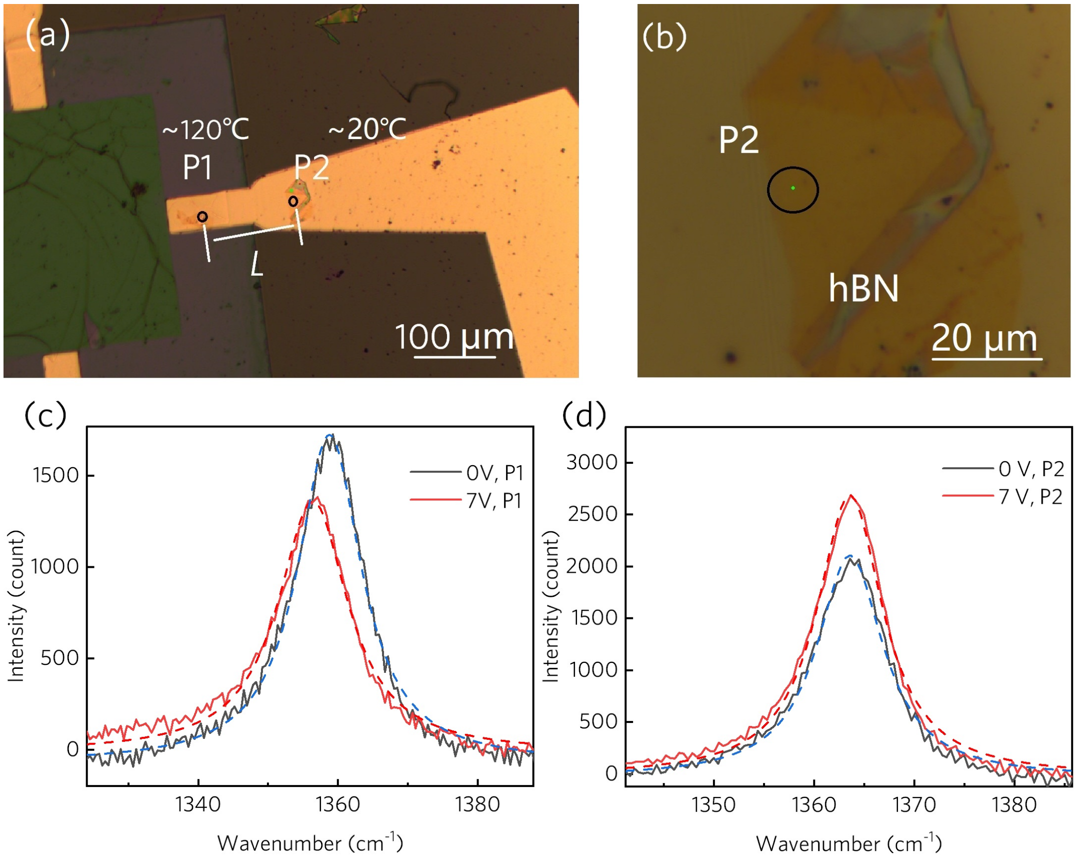

FIG. S12. Top left: Micrograph of a part of the test sample showing the measurement points P1 and P2 marked with black circles where the Raman shifts were recorded. Top right: close up of the region around P2 showing the edges of the hBN flake. Bottom left: Raman signal recorded at P1 on the membrane without and with voltage applied. Bottom right: The same for P2 on the frame. The shift at P1 is $3\,\mathrm{cm}^{-1}$ and at P2 is $< 0.1\,\mathrm{cm}^{-1}$. The voltage 7V corresponds to the power of 5.6 mW.

of the SiN membrane. The highest temperature is concentrated on both electrodes, reaching approximately 130 ℃, while the lowest temperature of about 70 ℃ is found in the middle of the membrane along the marked dash-line. In terms of deformation, the two electrodes deflect upwards, while the membrane close to the electrode area deflects downwards.

In addition, we confirm the linear relationship between the power P and the temperature T of the M electrodes by varying the static voltage V_{DC} applied to them. Since the deformation of the right M electrode and its neighboring SiN membrane in Fig. S14 closely resembles that observed at $P = 1.10$ mW in our experiment, we estimate the proportionality coefficient between P and T to be approximately $98K/\mathrm{mW}$. Consequently, the temperature T under different power P, as simulated by COMSOL, can be estimated as:

$$T = T_0 + P \times 98K/\mathrm{mW}. \tag{S16}$$

The coefficient obtained from the COMSOL simulation deviates by less than 10% from the temperature predicted by the simplified model presented in the main text. The spatial distribution of temperature and deformation agrees well with our experimental results, providing strong support for our simplified theoretical analysis outlined in the main text.

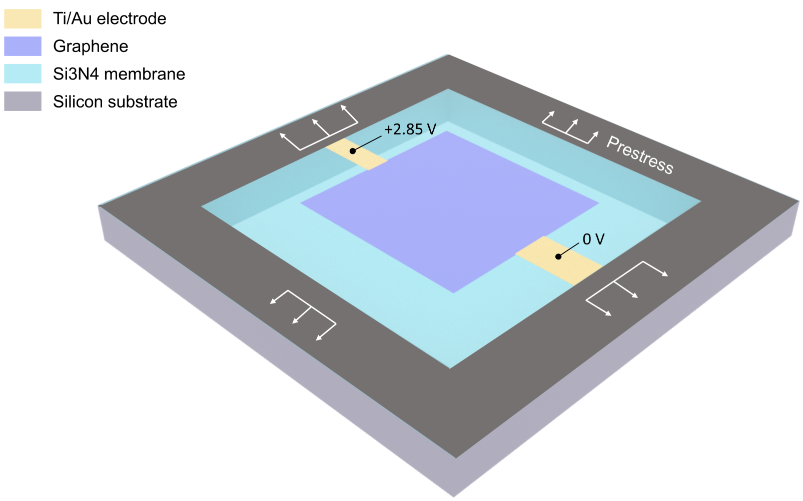

FIG. S13. Device characteristics and simulation setup

TABLE S2. Physics module setup

Physics module	Domains
Solid Mechanics	Graphene, Ti/Au electrode, SiN membrane, Si substrate
Electrical Currents	Graphene, Graphene, Ti/Au electrode, SiN membrane
Heat transfer in Solids	Graphene, Ti/Au electrode, SiN membrane, Si substrate

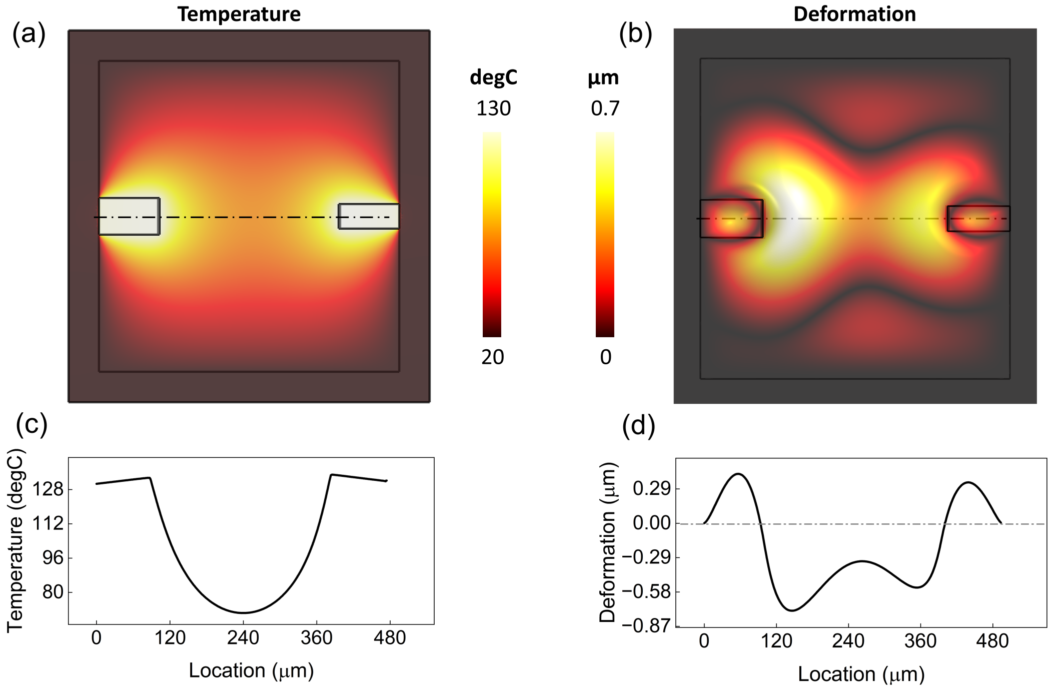

FIG. S14. The temperature distribution and deformation of the SiN membrane. Panels (a) depict the temperature distribution of the membrane from a top view. Panel (c) displays the temperature profile along the path between the two electrodes, marked by the dashed line. The deformation of the membrane is represented in panels (b) from a top view. The panel (d) exhibits the deformation profile along the path between the two electrodes, marked by the dashed line.

Destructive test: conductance and static deformation

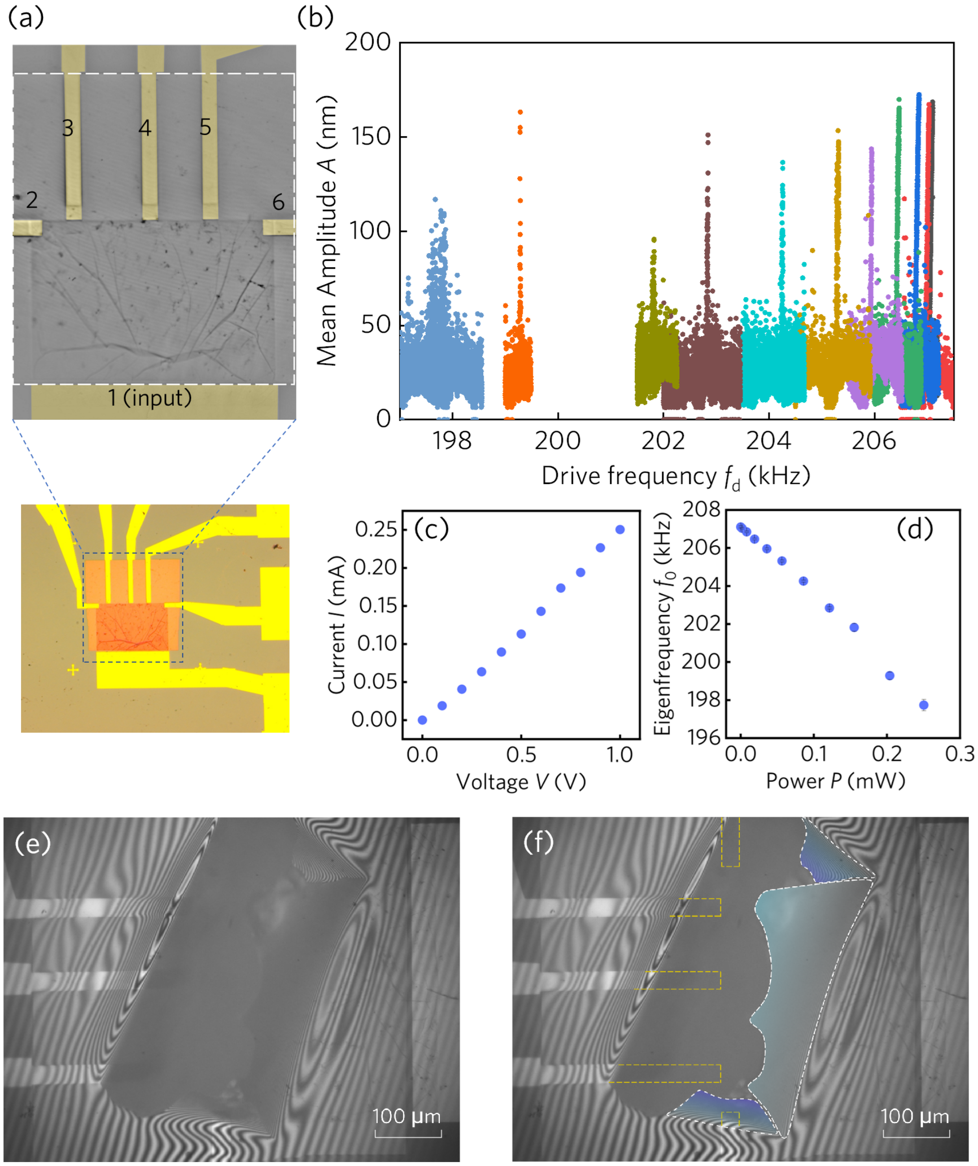

FIG. S15. DC voltage tuning of the eigenfrequency of the (1,1) mode of the resonator. (a) Light microscope image of the sample and a zoom of the sample (framed by the blue dashed line) captured by IWLI. The SiN membrane area is framed by the white dashed line. (b) IWLI measured amplitude response curves of the (1,1) mode under different input V_{DC}. (c) The two-point I-V between lead 1 and all others used together as counter electrode. (d) The relation of I^2 (proportional to the input power) and f_0. (e) Static interference picture of the sample captured by IWLI after the application of large V_{DC} to lead 1 w.r.t. all others on ground potential and continuously increasing it up to 7.0 V, until the membrane was destroyed. Figure rotated counterclockwise by 90° w.r.t. (a). (f) Same image as in (e) with markups of the boundary of the broken membrane (white dashed line). Areas of different pieces of the broken membrane are colored to indicate the most strained area. The yellow dashed lines mark the position of the electrical leads.

We here demonstrate a destructive test on a similar sample, a G-covered SiN prestressed membrane, with 6 electrodes on the surface of the SiN membrane, as shown in Fig. S15 (a), captured by IWLI. The only difference to the sample used in the main text is the geometry of the patterned G and M leads. The white dashed line indicates the boundary of the SiN membrane; the rectangle area with wrinkles corresponds to the patterned G and the yellow colored structures indicate the M leads, labeled from 1 to 6. For a better view, the optical image of the sample under the microscope is shown in Fig. S15 (c). The V_{DC} is applied between the electrode 1 and the others (2, 3, 4, 5, and 6 on the same ground

potential). A linear eigenfrequency shift can be observed while V_{DC} increases from 0.0 V up to 1.0 V in steps of 0.1 V. The resonance curves are captured by IWLI at different drive frequency as shown in (b) and the measured current and the fitted f_0 for corresponding V_{DC} are plotted in (d) and (e) as blue dots. The linear dependence is fitted to the I-V curve and I^2 - f_0 relation, as blue dashed line plotted in Fig. S15 (d) and (e). The I-V and the corresponding f_0 shifts due to the heating show similar behavior compared to the sample shown in the main text. However, when we increase V_{DC} up to around 6 V, the leads 2, 3, 4, 5 and 6 deform strongly, the leads drag the membrane down and finally break the membrane when V_{DC} reaches 7 V. The captured image after the membrane breakdown is shown in Fig. S15 (e). In panel (f) we painted the broken boundary with different transparent colors for highlighting the shape.

On-surface actuation of a MGS resonator

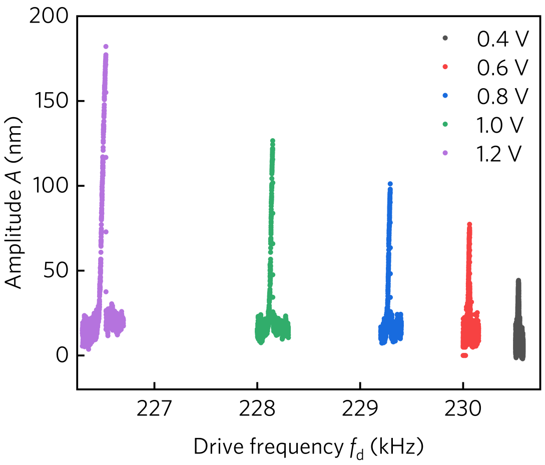

FIG. S16. Measured vibrational response curves of the (1,1) mode as function of the drive frequency. Same data as presented as function of the detuning in Fig. 4 in the main text.

Different V_{AC} are applied between leads 1 and 4. We sweep the frequency of V_{AC} close to the eigenfrequency of the (1,1) mode, hence we excite the membrane into vibration. We here show the same data as shown in Fig. 4 in the main text, but now plotted as function of the drive frequency. With increasing V_{AC} the effective power dissipated between leads 1 and 4 produces an overall heating effect which leads to a red shift of f_0. The maximum amplitude response of the driven mode increases while increasing the intensity of V_{AC}.

Control experiments

(1) Simple metal leads on top of the SiN membrane. The system can be regulated by heating the metal leads (see Fig. S17 (a)). To maximize the tuning effect, it is necessary to introduce more heating power, increasing the number, thickness or length of the leads would help. In any case a trade-off is necessary between the required space on the surface and the geometry of the leads to provide sufficient resistance and avoiding breakdown. Here, probe A (length: 570 μm, width: 4 μm and thickness: 27 nm) on the membrane was used to load the heating power. As showing in Fig. S17 (b), the power was loaded from 0 mW up to approximately 0.6 mW, the eigenfrequency of the fundamental mode shows a shifting of 13 kHz corresponding to a 5.2% tuning capability. However, to prevent breakdown, we cannot further increase the heating power, because the I-V relation of the electrode becomes nonlinear when the power is increased to approximately 0.6 mW. Our findings suggest that the regulation capability of simple metal leads is much less than the one of our MGS structure.

(2) Based on trial (1), we designed Al-AlO_x-Al composite structures, as shown in Fig. S17 (c). Each unit of the regulation structure is composed of a top Al electrode, a bottom Al electrode and a layer of very thin AlO_x in between. The atomic AlO_x layer is fabricated by gently oxidizing the surface of the bottom Al electrode after evaporation. The atomic AlO_x layer determines the resistance of the Al-AlO_x-Al composite structures. However, the experiments show

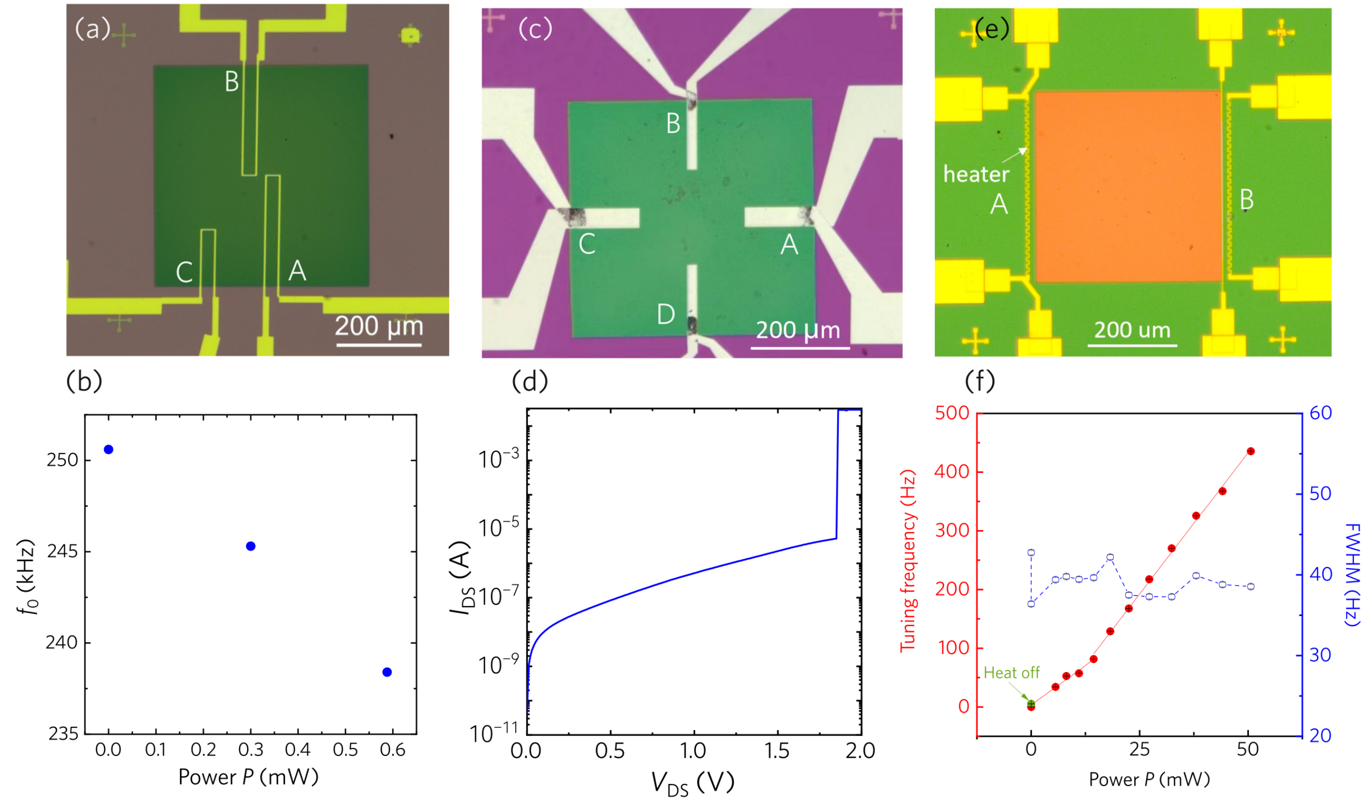

FIG. S17. Trial samples for the control experiments. (a) Al metal leads on top of SiN membrane. (b) The frequency tuning measured on the lead A of the sample shown in panel (a). (c) Al-AlO_x-Al composite structures of top of SiN membrane. (d) the measured I-V curve of the lead A on sample shown in panel (c). (e) Ti/Au metal heaters on top of frame (out of the SiN membrane). (f) The frequency tuning measured on the heater A of the sample shown in panel (e).

that such atomic AlO_x layer is not very robust against electric breakdown. The breakdown of the leads is indicated by the black fuzzy area (close to the frame) on all four Al-AlO_x-Al electrodes. The recorded typical I-V relation is shown in Fig. S17 (d). For the sample shown in Fig. S17 (c), the breakdown voltage was in the order of 1 V, and the frequency tuning was negligible.

(3) Based on our previous investigations, we noticed that when generating the heat on the frame, the eigenfrequency will be tuned slightly up in contrast to the observations in our MGS structure. This is caused by the increasing tensile stress of the membrane between the mismatch of the thermal expansion coefficient of SiN and Si under increasing temperature difference. Thus, we fabricated a test sample with metal leads (Ti/Au with thickness 30 nm) on the frame as shown in Fig. S17 (e). Again, the tuning capability is limited (less than 0.5‰ in the presented case in Fig. S17 (f)). Moreover, this control experiment also indicates that to maximize the tuning capability, it is important to generate the heat in a localized area on the membrane.

Applications of MGS devices

(1) Micromirror

MEMS/NEMS mirrors commonly include two parts: a suspended membrane with a metal layer on top of it and its MEMS actuator. The main parameters for the MEMS mirrors are angular range, power consumption and response time.

To use the MGS device as MEMS mirror, one pair of MGS structure is needed as shown in Fig. 1 in the main text. Note that we do not use the tuning effect of the eigenfrequency in this application. The metal electrode deposited on the membrane provides the bending area as the mirror for reflecting laser beam. For applying a DC voltage, we can statically change the reflecting angle, and by applying an AC voltage, we can use it as a scanning mirror for LIDAR

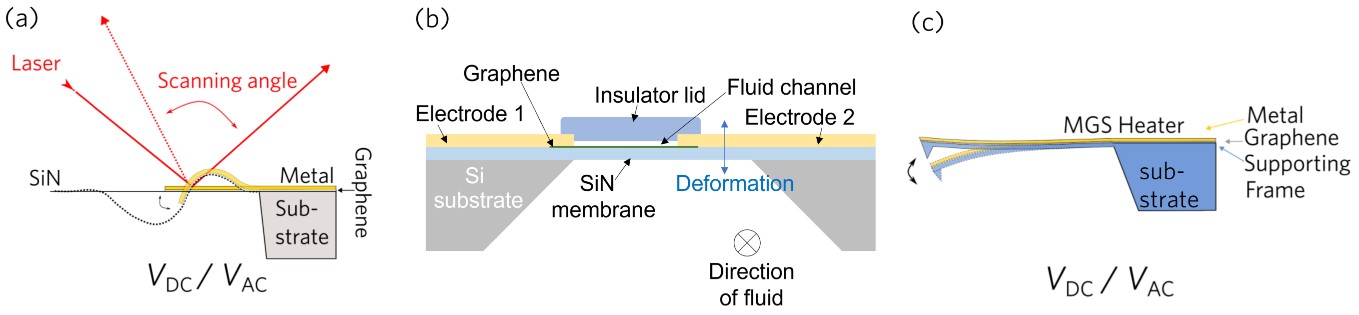

FIG. S18. Possible applications of MGS devices. (a) Sketch for a demonstration of a micro-mirror (scanning mirror / galvometer) design. The cross-section of the scanning mirror, which indicates the scanning process by using an AC voltage. The scanning angle can be controlled by the intensity of the AC signal. (b) Sketch for a demonstration of a microcontroller for fluid. (c) The sketch indicates the active AFM tip design by using a MGS structural as heater component. With applying an AC voltage, the AFM tip can perform the tapping mode. Controlling of a applied DC voltage, the AFM tip can be approached in nanometer steps.

application as shown in Fig. S18 (a). Another metal electrode can be even located on the frame so that no bending occurs, simplifying the deformation of the working area after current injection.

The MGS devices show several advantages in the application of MEMS mirrors. First, in principle the device has simpler structure and ability to be further scaled down, since the MGS device is used as both the metal mirror and its MEMS actuator. Second, the response time of MGS device is very fast. This can be known from our on-chip excitation experiments with eigenfrequency around 200 kHz. For this application, low Q factors and operation at damping close to the critical value for overdamping is advantageous to reduce unwanted oscillations of the mirror during state transitions. Third, the power consumption is low as shown in our manuscript compare to a typical micromirror [S11]. The power consumption can be further reduced by lowering the prestress of the SiN membrane, as discussed in our model for the threshold voltage of inducing deformation. The quickly controllable MEMS mirrors currently are highly demanded in micro-optical systems.

(2) Miniature mechanical switches or controllers

The concept of controller for the microfluidic is similar to the mirror but a lid or sidewalls for the fluid channel is necessary in addition to the MGS structure, as shown in Fig. S18 (b). A fluid channel with indeformable sidewalls is placed on the Si frame without suspended SiN membrane and leads the fluid to the microcontroller. When there is no heating on the MGS structure, the gap between the insulator lid and SiN membrane is very small and thus the flow of the fluid is low, which is the ‘off state’. After the MGS structure is heated and the SiN membrane is deformed, the fluid channel in the microcontroller becomes larger and the flow becomes much larger, which is the ‘on state’.
Since our MGS structure has a short response time, the controller also can switch quickly. Besides, by using multiple electrodes we can fabricate more than one microcontroller on one SiN membrane.

(3) Active AFM tip

The AFM cantilever can be deformed by the metal heater structure on top. To introduce as much heat as possible, the metal structure has to be wired as long as possible but will be limited by the width of the wire, i.e., the breakdown voltage. With the optimization of the MGS structure as a new heater component, as demonstrated in Fig. S18 (c), the limitation will be significantly released. Proved by our experiments in the manuscript, the bending will be strong and can survive under higher voltage. With applying an AC voltage, the AFM tip can perform the tapping mode, the response will be fast enough for the tapping. By applying a DC voltage, the AFM tip can be approached in nanometer steps.

For a short summary, this design scheme can be very efficient on all possible MEMS/NEMS devices which require actuation with free-standing structures, such as gyroscope, inertial sensor, membrane pressure sensor, and so on.

---

* fan.yang@uni-konstanz.de

[S1] B. Bošnjak, *Investigating the geometry dependency of modulated graphene by ESR measurements*, Master’s thesis, Universität Hamburg (2017).

[S2] F. Yang, M. Fu, R. Waitz, and E. Scheer, *Quantitative signal extraction in the dynamic range of nanomechanical systems by free and constrained fitting*, Sensors and Actuators A: Physical **354**, 114307 (2023).

[S3] R. Waitz, S. Nößner, M. Hertkorn, O. Schecker, and E. Scheer, *Mode shape and dispersion relation of bending waves in thin silicon membranes,* Physical Review B **85**, 035324 (2012).
[S4] R. Waitz, C. Lutz, S. Nößner, M. Hertkorn, and E. Scheer, *Spatially resolved measurement of the stress tensor in thin membranes using bending waves,* Physical Review Applied **3**, 044002 (2015).
[S5] S. Petitgrand, R. Yahiaoui, K. Danaie, A. Bosseboeuf, and J. Gilles, *3d measurement of micromechanical devices vibration mode shapes with a stroboscopic interferometric microscope,* Optics and Lasers in Engineering **36**, 77 (2001).
[S6] H. Chen, H. Jia, C. A. Zorman, and P. X.-L. Feng, *Determination of elastic modulus of silicon carbide (sic) thin diaphragms via mode-dependent duffing nonlinear resonances,* Journal of Microelectromechanical Systems **29**, 783 (2020).
[S7] C. Liu, Z. Zheng, X. Yang, and J. Guo, *Geometric nonlinear vibration analysis for pretensioned rectangular orthotropic membrane,* International Applied Mechanics **54**, 104 (2018).
[S8] A. A. Ramadan, R. D. Gould, and A. Ashour, *On the van der pauw method of resistivity measurements,* Thin Solid Films **239**, 272 (1994).
[S9] S.-a. Peng, Z. Jin, P. Ma, D.-y. Zhang, J.-y. Shi, J.-b. Niu, X.-y. Wang, S.-q. Wang, M. Li, X.-y. Liu, *et al.*, *The sheet resistance of graphene under contact and its effect on the derived specific contact resistivity,* Carbon **82**, 500 (2015).
[S10] S. Timoshenko, *Analysis of bi-metal thermostats,* Journal of the Optical Society of America **11**, 233 (1925).
[S11] B. R. Samanta, F. Pardo, T. Salamon, R. Kopf, and M. S. Eggleston, *Low-cost electrothermally actuated mems mirrors for high-speed linear raster scanning,* Optica **9**, 251 (2022).
